# Organ Specific Proteomic Dissection of *Selaginella bryopteris* Undergoing Dehydration and Rehydration

**DOI:** 10.3389/fpls.2016.00425

**Published:** 2016-04-08

**Authors:** Farah Deeba, Ashutosh K. Pandey, Vivek Pandey

**Affiliations:** Plant Ecology and Environmental Science, CSIR-National Botanical Research InstituteLucknow, India

**Keywords:** *Selaginella bryopteris*, dehydration, rehydrations, root, frond, 2 dimensional gel eletrophoresis, MALDI/TOF-TOF

## Abstract

To explore molecular mechanisms underlying the physiological response of *Selaginella bryopteris*, a comprehensive proteome analysis was carried out in roots and fronds undergoing dehydration and rehydration. Plants were dehydrated for 7 days followed by 2 and 24 h of rehydration. In roots out of 59 identified spots, 58 protein spots were found to be up-regulated during dehydration stress. The identified proteins were related to signaling, stress and defense, protein and nucleotide metabolism, carbohydrate and energy metabolism, storage and epigenetic control. Most of these proteins remained up-regulated on first rehydration, suggesting their role in recovery phase also. Among the 90 identified proteins in fronds, about 49% proteins were up-regulated during dehydration stress. Large number of ROS scavenging proteins was enhanced on dehydration. Many other proteins involved in energy, protein turnover and nucleotide metabolism, epigenetic control were also highly upregulated. Many photosynthesis related proteins were upregulated during stress. This would have helped plant to recover rapidly on rehydration. This study provides a comprehensive picture of different cellular responses elucidated by the proteome changes during dehydration and rehydration in roots and fronds as expected from a well-choreographed response from a resurrection plant.

## Introduction

The response of plants to drought has economic implications directly affecting plant productivity. Based on predictions of global environmental changes, it is proposed that developing drought tolerant crops while maintaining productivity will become a critical requirement in the early part of Twenty First century (Ramanathan, 1988). Understanding how plants tolerate water loss is a vital pre-requisite for developing drought tolerance and biomass/seed production of plants under drought conditions.

Most of the flowering plants are drought sensitive and cannot survive if the water content falls below 59–30% although dehydration is an integral part of the normal developmental program of higher plants in the context of seed formation. Only a few plants possess dehydration tolerant vegetative tissues; these include a small group of angiosperms, termed resurrection plants (Gaff, 1971), some ferns (Farrant et al., 2009), algae (Holzinger and Karsten, 2013), lichens (Beckett et al., 2005), and bryophytes (Cui et al., 2012). Mature tissue of resurrection plants such as leaves and roots are able to remain in the air-dried state for months by reaching a quiescent state which is comparable with dormancy in seeds in several aspects (Bartels, 2005). Resurrection plants have the ability.

Drought stress affects both the underground and above ground structures such as roots or leaves, triggering cellular signal transduction pathways for molecular and metabolic changes. Hence it is important to study both root and leaf systems together for better understanding of how plants respond to drought stress. Proteins associated with the primary function of an organ, are uniquely expressed in specific organ/tissues (Watson et al., 2003). This organ specific expression of protein is thus essential for plant growth and development. Organ-specific proteomic analyses help in better understanding the response mechanisms of plants toward drought stress.

Proteomics is a link between genomics, genetics and physiology (Zivy and de Vienne, 2000) since it provides a more physiologically accurate snapshot of biochemical processes by revealing the actual protein constituents performing the enzymatic, regulatory, and structural functions encoded by the genome and transcriptome at a given point in time. Thus, proteomics has become an essential technique to study plant drought-resistance mechanisms with respect to large-scale analysis of proteome variations (Cooper and Farrant, 2002; Ingle et al., 2007; Carpentier et al., 2008; Delaplace et al., 2009). Two dimensional gel electrophoresis along with mass spectrometry is a powerful approach for identifying drought responsive proteins. It has been reported that *Selaginella bryopteris* overcomes the drought induced mechanical, oxidative and destabilizing stress by relying on morphological adaptation (leaf curling), antioxidant protection (SOD, CAT, APX), accumulation of proline etc. (Pandey et al., 2010). Proteomic studies suggest that multiple metabolic processes are involved in dehydration response and tolerance (Dinakar and Bartels, 2013). In an earlier study on detached fronds of *S. bryopteris*, we found higher expression of protein related to protein synthesis and degradation (Deeba et al., 2009). Wang et al. (2010) identified 103 unique desiccation responsive proteins in *S. tamariscina*. These proteins were mainly involved in photosynthesis, carbohydrate and energy metabolism, stress and defense, signaling, cell structure and cell division. Expressed Sequence Tags (EST) analysis of *S. lepidophylla* has shown that genes involved in transport, cell structure, secondary metabolism, protein modification etc. account for a large portion of genome (Iturriaga et al., 2006). However, all the studies have been carried out in fronds of *Selaginella*. There is no report on effect of dehydration and rehydration on roots of this unique plant. The objective of the present study was to identify proteome wide changes in both roots and fronds of *S. bryopteris* to obtain a more compehensive picture of the proteins that are involved in dehydration tolerance and rehydration. To our knowledge, this is the first report of proteomic analysis of *S. bryopteris* roots and fronds under dehydration and rehydration.

## Experimental section

### Plant material

The plants of *Selaginella bryopteris* were collected from Mirzapur district situated in the west of Uttar Pradesh (latitude 23°52′−25°32′N and longitude 82°7′−83°33′E). Plants were maintained in pots containing neopeat planting material mixed with garden soil and kept them in fern house for acclimatization under natural sunlight with PPFD <1000 μmol^−2^s^−1^ and 60–70% of humidity. The plants showed better growth during spring and monsoon seasons (February to April and July to September). However, fronds turned brownish and curled inward during the peak summer and winter seasons.

### Experiments of dehydration and rehydration in *Selaginella bryopteris*

All the experiments were conducted in growth chamber (Conviron, PGR-15, Canada). Healthy *Selaginella* plants were allowed to dry for 7 days by withholding water at 25°C and <20 μmol m^−2^ s^−1^ PPFD (maintaining a diurnal rhythm of 13 h day and 11 h dark cycle) until the photochemical efficiency of PSII (*F*_v_/*F*_m_) reached to its minimum and remained stabilized at this point. After 7 days of dehydration, the fronds were rehydrated till fronds were fully opened. Altogether we have taken four points of sampling *a*. control, *b*. dehydrated samples (DE), *c*. rehydrated sample 2 h after rehydration (RI) and *d*. rehydrated sample after 24 h (RII) until *F*_v_/*F*_m_ reached to its original values. All the samples were collected between 9 and 11 am to avoid apparent differences in protein abundance caused by circadian or light dark regulation. At every sampling point the proteins of roots as well as fronds were extracted and differential proteomic analyses were done. Three independent biological replicates were taken for each treatment.

### Isolation of root and frond proteins and two-dimensional gel electrophoresis

Proteins for each treatment (DE, RI, and RII along with control) in *Selaginella* roots and fronds were extracted according to the modified method (Damerval et al., 1986). The roots and fronds of *S. bryopteris* were collected randomly each from independent biological replicate and were pooled together for further analysis. Samples were ground in liquid N_2_ and the resulting powder was extracted with 0.05 M Tris-HCl pH 8.0, 0.025 M EDTA, 0.5 M thiourea and 0.5% β-mercaptoethanol. The extract was mixed with 10% cold TCA and 0.07% BME, and left overnight at −20°C. The mixture was centrifuged at 4500 rpm for 10 min and the pellet was washed three times with 10% acetone and 0.07% BME. The pellet was then vacuum dried, solubilized in 0.1 M Tris HCl, pH 8.0, 0.05 M EDTA and 2% BME. Proteins were then extracted with 2.5 mL Tris- buffered phenol and centrifuged at 4500 rpm for 10 min. After centrifugation, lower phenol phase was collected with the help of Pasteur pipette. To this 10 ml 0.1 M ammonium acetate in methanol was added and left overnight at −20°C.

The mixture was centrifuged at 4500 rpm for 10 min and pellet was dissolved in 0.1 M ammonium acetate in methanol and 1% BME. It was centrifuged at 6000 rpm for 10 min and was washed twice with cold acetone. Dried pellet was re-suspended in a solubilization buffer consisting of 7 M urea, 2 M Thiourea, 0.5% CHAPS, 0.02 M DTT, and 0.5% v/v immobilized pH gradients buffers. The total protein concentration was quantified by the Bradford assay (Bio-Rad, Hercules, CA, USA) with BSA as the standard.

Two-dimensional electrophoresis (2-DE) was carried out with some modifications (Lehesranta et al., 2005). Immobilized pH gradient (IPG) strips (GE Healthcare, 7 cm, pH 4-7, linear) were rehydrated overnight with 135 μl of rehydration buffer (7 M urea, 2 M Thiourea, 2% CHAPS, 0.02 M DTT, 0.5% v/v immobilized pH gradient buffers) containing 35 μg protein (for Sypro ruby staining) or 120 μg (for commassie staining) in a reswelling tray (Amersham Biosciences, Uppsala, Sweden) at room temperature. Isoelectric focusing (IEF) was conducted at 20°C with an Ettan IPGphore-3 (GE Healthcare).

The focusing conditions were as follows: 250 V for 30 min, 450 V for 15 min, 750 V for 15 min, and 2000 V for 30 min and 8000 V for 2 h for a total of 15 kVh. The focused strips were equilibrated twice for 15 min in 10 ml of equilibration solution. The first equilibration was performed in a solution containing 6 M urea, 30% w/v glycerol, 2% w/v sodium dodecyl sulfate (SDS), 1% w/v DTT and 50 mM Tris-HCl buffer, pH 8.8. The second equilibration was performed in a solution modified by the replacement of DTT by 2.5% w/v iodoacetamide. For SDS-PAGE, the equilibrated strips were positioned on the stacking gel and sealed with 0.5% agarose solution. The second dimension was run in Hoefer mini-gel apparatus in 7 × 8 cm homogeneous 12% SDS PAGE gels. Electrophoresis was performed in a standard Tris-Glycine running buffer at a constant voltage of 200 V. The analytical gels were stained with Sypro ruby (Invitrogen) and preparative gels were stained with coomassie brilliant blue G (Sigma Aldrich). Three technical replicated were run for each biological replicates in roots and fronds of *S. bryopteris* (Supplementary Information 1).

### Image acquisition and data analysis

The gel images were acquired with the typhoon™ 9200 scanner (GE Healthcare, USA). The data were analyzed using Image Master 2D Platinum 7.0 software™ (GE Healthcare, USA). The gels were taken in triplicate for each treatment and all gels were detected for their spots by taking the parameters of smoothness as 2, minimum area as 5 and saliency as 2. Relative volume (% volume) was used to quantify and compare the spots. Relative volume considers the ratio of detected spot pixel density to the sum of all analyzed spot pixel density. Hence, this procedure permitted to normalize experimental variations due to protein loading and staining. The criteria for defining the protein expression were taken as 1.5 fold increase or decrease during the treatments. A criterion of *p* < 0.001 was used to define the significant difference when analyzing the parallel spots between groups with analysis of one-way variance (ANOVA).

For each treatment, at least three 2-DE gels, representing three biological replicates, were used for data analysis. The spots were used to calculate mean value for a given spot, and this value was used as the spot quantity on the standard gel (Supplementary Datasets S1, S2 in Supplementary Information 2).

### Protein identification

Tryptic digestion of the protein spots excised from the gels, and sample preparation were performed (Koistinen et al., 2002). Gel particles were destained overnight by 50% methanol and 0.05 M ABC. Next morning, gels were re-swelled by replacing destain solution with sterilized MQ water for about 5–8 min and fresh volume of destain solution were added for upto 3–4 h. Gels were washed twice with 0.025 M ABC for 10 min and dehydrated by washing with 2:1 solution of ACN and 0.05 M ABC.

The cycle of dehydration was followed by rehydration by 0.025 M ABC three times. Destained gel pieces were dried in a vacuum centrifuge concentrator for 30 min and dried gel pieces were rehydrated in trypsin solution (10–20 μl from 20 ng/μl trypsin stock solution) which were added according to 1:20 ratio of protein. Gel particles were immersed in 0.025 M ABC and samples were digested overnight at 37°C (about 16–18 h). Peptides were extracted twice with 50% ACN/1% TFA. The recovered peptides were concentrated to a final volume of 10 μl. The database search criteria were as follows: taxonomy, viridiplantae, peptide tolerance, ±100 ppm, MS/MS tolerance, ±0.2 Da; peptide charge +1; maximum allowed missed cleavage, 1; fixed modification, cysteine carbamidomethylation; variable modification, methionine oxidation; instrument type, MALDI-TOF/TOF. Protein scores were derived from ion scores as a non-probabilistic basis for ranking protein hits and as the sum of the series of peptide scores. The score threshold to achieve *p* < 0.05 was set by the mascot algorithm and was based on the size of the database used in the search. False discovery rate (FDR) for identification was set to 1%.We considered only those protein spots whose MOWSE score was above the significant threshold level determined by Mascot. Proteins with the confidence interval percentage of greater than 95% were considered to represent a positive identification and were also evaluated on the basis of various parameters, such as the number of peptides matched, and % coverage of matched protein. In all the protein identifications, probability scores were greater than the score fixed by Mascot as significant with a *p* < 0.05 (Supplementary Datasets S1, S2 in Supplementary Information 3). Some of the MS/MS spectra of samples were identified by using ProteinPilot software 1.0 (Protein Pilot software v. 4.0, rev. 148085; Applied Biosystems, Foster City, CA, USA) with the Paragon search engine. The default search settings used for protein identification were: enzyme, trypsin; Cys alkylation, iodoacetamide; special factor, gel-based ID; and ID focus, biological modification and amino acid substitution. We report only protein identifications with a total ProtScore >1.3, which represents >95% statistical confidence in Protein Pilot (Yang et al., 2007; Alvarez et al., 2009). Protein sequences that were identified as “unknown” or as “hypothetical protein,” were further annotated by using the protein homologs sequences for an additional query using BLASTP algorithm (http://blast.ncbi.nlm.nih.gov/Blast.cgi), searching first the UniProtKB/Swiss-Prot database, and then the NCBI non redundant database. For the total number of observed peptides per protein, the unique sequences were counted and were imported to Microsoft Excel (Supplementary Datasets S3, S4 in Supplementary Information 4).

### One-way ANOVA analysis

A criterion of *p* < 0.001 was used to define the significant difference when analyzing the parallel spots between groups with analysis of one-way variance (ANOVA) on the treatment specific expression values of both *S. bryopteris* root and fronds taking into consideration the three treatments to identify significantly changed proteins expression (Supplementary Information 2; Tables S1, S2). A principal component analysis was performed on log10-transformed dataset (Pareto-scaled) using Simca P+ software (12.0.1, Umetrics, Umeå, Sweden).

## Results

**Figure 1:**
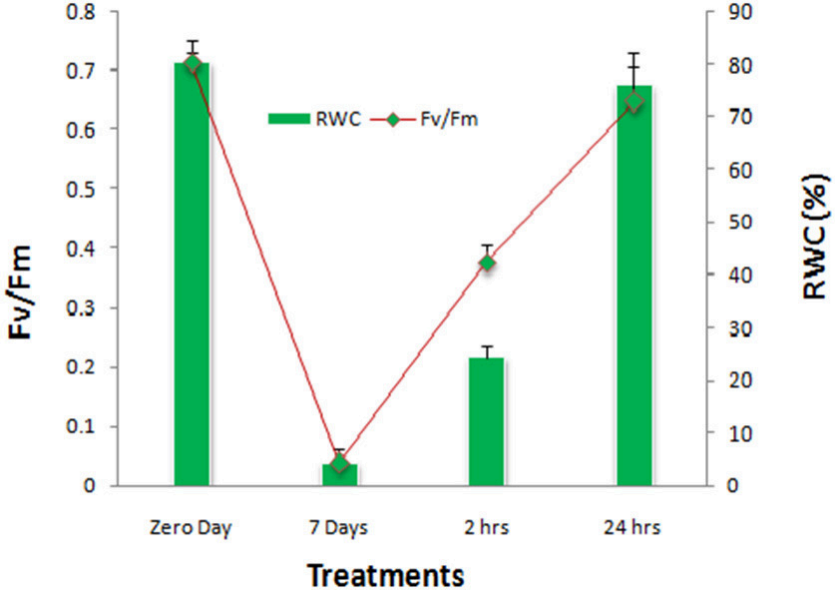
**Photochemical efficiency of PS-II (F_**v**_/F_**m**_) and relative water contents (RWC) of ***S. bryopteris*** during dehydration and rehydration**.

In the course of dehydration, the aerial parts of plants exhibited frond rolling and wilting. Gradually, the plants curled up and the crown decreased. In fully dehydrated condition the curled fronds showed 4.25% water content (Figure 1). When water was provided again, the aerial parts initially partially opened after 2 h (RI) and fully opened after 24 h (RII). The effect of drought on relative water content (RWC) and photochemical efficiency of PSII (*F*_v_/*F*_m_) reflected the negative effects on both the parameters. With the significant decrease in RWC by 94%, the *F*_v_/*F*_m_ was also concomitantly declined by 94% which was found to be recovered by 53% after RI followed by almost 90% recovery on RII (Figure 1).

### *S. bryopteris* root proteomics

**Figure 2:**
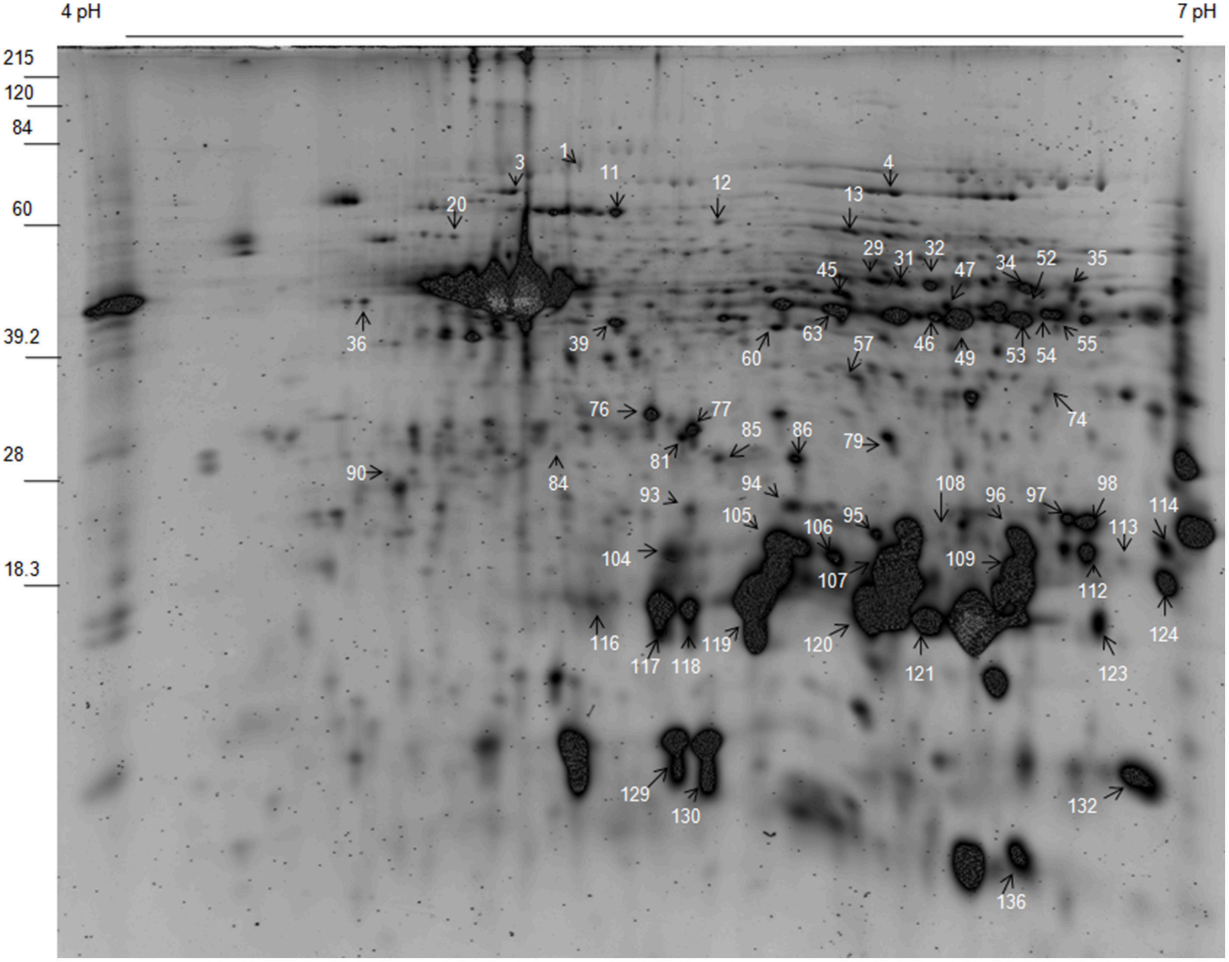
**Representative gel of ***S. bryopteris*** root proteins**.

In *Selaginella* roots, more than 730 protein spots were detected, out of which, 548 spots were matched to all the treatment gels, and 136 spots were found to be differentially expressed out of which 59 spots were identified (Figure 2, Table 1, Table S1 in Supplementary Information 6). These proteins were analyzed by peptide mass fingerprinting (PMF) and MS/MS using MALDI-TOF-TOF. In roots, barring one, all the proteins were significantly up-regulated during dehydration. The major proteins belonged to the categories of nucleotide metabolism (7 proteins; Table 1), stress and defense (7), carbohydrate and energy metabolism (6) and signaling (5) (**Figure 4**).

**Table 1:** **List of differentially expressed proteins in the roots of ***S. bryopteris*** during dehydration (DE) and on rehydrations (RI and RII)**.

**Spot ID**	**Identified proteins**	**Accession no**.	**Folds changes in protein expression**			**Peptide matched**	**Sequence coverage (%)**	**Theort PI/MW**	**Observed pI/MW**
			**DE**	**RI**	**RII**				
**SIGNALING**									
13	Similar to *S. cerevisiae* PTR2 gene, GenBank Accession Number L11994 [*Arabidopsis thaliana*]	gi|575427	2.3 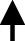	3.4 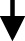	1.4 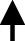	2	1	5.2/68	6.0/69
57	Short-chain dehydrogenase, putative [*Ricinus communis*]	XP_002531343.1	2.7 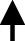	2.0 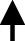	1.3 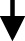	1	4	9.9/23	6.1/38
84	Phosphatase 2C family protein [*Populus trichocarpa*]	gi|224063237|XP_002301055.1	3.6 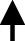	3.0 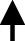	1.5 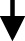	3	11	6.7/30	5.4/24
90	14-3-3d protein [*Gossypium hirsutum*]	gi|164652940	2.1 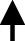	1.5 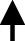	–	1	6	4.7/29	4.7/28
106	PREDICTED: probable LRR receptor-like serine/threonine-protein kinase At1g29720-like [*Vitis vinifera*]	gi|359483557	4.4 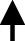	3.3 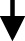	–	1	1	6.4/111.7	6.0/21
**MEMBRANE TRANSPORT**									
77	ATP-binding cassette transporter, subfamily C, member 1, cluster I, SmABCC1 [*Selaginella moellendorffii*]	XP_002964599.1	2.2 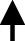	1.9 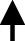	1.6 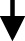	1	3.6	8.3/177	5.6/33
124	DMI1 protein [*Physcomitrella patens*]	ABC70463.1	2.7	1.8 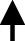	–	2	5.7	5.4/75	6.9/18
**STRESS AND DEFENSE**									
34	ALDH11A3 [*Arabidopsis lyrata* subsp. lyrata]	gi|297825375|XP_002880570.1	1.5 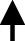	1.6 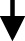	2.0 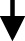	4	9	7.0/53	6.5/51
35	Tau class glutathione S-transferase [*Pinus tabuliformis*]	AAT69969.1	2.7 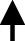	1.9 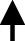	1.3	6	17.1	6.2/25	6.6/50
81	Lactoylglutathione lyase (*Ricinus communis*)	XP_002514254.1	1.6 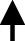	2.2 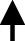	1.2 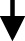	8	14	5.3/32	5.6/36
107	Glutathione S-transferase-like protein [*Solanum lycopersicum*]	gb|AAL92873.1|NP_001234157.1	2.0 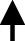	1.5 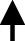	1.5 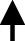	1	3	6.2/25	5.9/20
113	Thioredoxin-like protein [*Arabidopsis thaliana*]	gb|AEE30092.1||	2.6 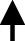	1.7 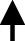	–	3	9	7.8/19	6.8/20
114	Serine carboxypeptidase family protein [*Hyphomonas neptunium* ATCC 15444]	gb|ABI76221.1|	2.7 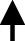	1.8 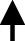	–	1	3	9.4/52	6.9/22
136	Leucine-rich repeat family protein [*Arabidopsis lyrata* subsp. lyrata]	XP_002873330.1	2.6 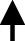	1.8 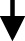	1.4 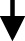	1	6.3	8.6/28	6.4/8
**CELL WALL**									
45	PREDICTED: phospholipase A1-IIgamma-like [*Solanum lycopersicum*]	XP_004232966.1	1.8 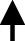	1.4 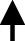	–	1	93	5.1/44	5.8/48
60	Glucan endo-1,3-alpha-glucosidase Agn1 [*Schizosaccharomyces japonicus yFS275*]	XP_002174591.1	1.2 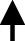	–	1.6 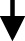	1	2	4.8/51	5.8/45
**PROTEIN METABOLISM**									
3	Peptide chain release factor 1 [*Arabidopsis thaliana*]	NP_182225.3	2.3 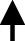	1.5 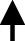	1.3 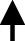	1	3.1	5.9/43	5.1/68
11	Hsc70 [*Solanum lycopersicum*]	gi|762844	2.0 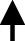	1.3 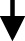	1.3 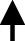	5	9	5.2/71	5.4/62
12	Heat shock protein, putative [*Ricinus communis*]	XP_002518324.1	1.7 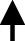	2.1 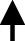	1.4 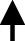	4	13.6	5.4/67	5.6/60
96	Aminoacyl-t-RNA synthetase [*Arabidopsis thaliana*]	gi|4678317|CAB41128.1	2.1 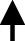	1.4 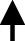	–	1	1	5.7/119	6.5/22
129	Ankyrin repeat-containing protein [*Arabidopsis thaliana*]	gi|15232175	1.5 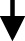	–	4.2 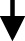	2	1	9.6/73	5.6/11
**CELL DIVISION, DIFFRENTIATION AND FATE**									
20	LAS1-like family protein [*Arabidopsis thaliana*]	NP_196783.2	1.4 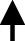	2.1 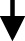	–	1	39	6.2/74	5.9/58
132	UBX domain-containing protein [*Arabidopsis thalian*a]	NP_567675.1	2.0 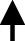	1.7 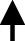	–	2	5.7	4.8/39	6.8/10
**NUCLEOTIDE METABOLISM**									
4	Nucleoside-triphosphatase/nucleotide binding protein [*Arabidopsis lyrata* subsp. lyrata] XP_002874350.1	XP_002874350.1	2.0 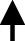	1.3 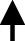	1.2 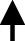	1	3.3	7.2/30	6.2/66
36	Putative DNA repair protein RAD23-1 [*Arabidopsis thaliana*]	NP_850982.1	1.6 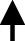	1.6 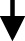	–	1	2	4.5/39	4.6/47
46	RNA binding protein, putative [*Ricinus communis*]	XP_002519274.1	1.9 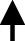	22.6 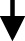	–	1	3.6	5.4/43	6.2/44
86	Pentatricopeptide repeat-containing protein [*Arabidopsis thaliana*]	NP_178983.1	2.0 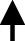	1.9 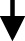	–	1	4.8	5.2/56	5.7/30
94	Pyrimidine-specific ribonucleoside hydrolase rihA [*Zea mays*]	ACG36517.1	1.9 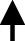	–	1.5 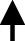	1	3	5.5/35	5.8/25
98	Pentatricopeptide repeat-containing protein [*Arabidopsis thaliana*]	NP_189568.1	1.9 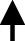	1.5 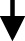	–	1	3	5.3/46	6.7/23
130	Nucleotidyltransferase family protein, putative, expressed [*Oryza sativa* Japonica Group]	gi|77548394|ABA91191.1	2.3 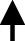	1.5 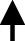	–	1	1	5.6/86	5.6/10
**CARBOHYDRATE AND ENERGY METABOLISM**									
29	Enolase [*Gossypium hirsutum*]	gi|158144895	2.6 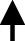	1.9 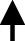	1.3 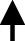	1	3	5.5/47	5.8/50
32	Enolase	gi|90110845	1.9 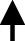	–	–	3	11	5.4/48	6.2/50
52	ATPase subunit [*Beta vulgaris* subsp. vulgaris]	gi|11263	5.6 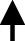	3.2 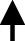	3.7 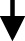	1	2	5.7/55	6.4/48
74	Glucose and ribitol dehydrogenase [*Medicago truncatula*]	XP_003591094.1	3.3 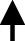	2.2 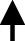	–	1	98	6.4/30	6.6/36
85	Ketose-bisphosphate aldolase class-II-like protein [*Arabidopsis thaliana*]	NP_173263.2	2.1 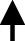	1.5 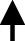	1.4 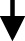	1	3	5.8/184	5.8/30
93	ATPase alpha subunit [*Selaginella uliginosa*]	ABI54717.1	2.4 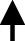	41.5 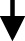	–	7	100	9.0/23	5.6/26
108	ATP-binding cassette transporter, subfamily C, member 1, cluster I, SmABCC1 [*Selaginella moellendorffii*]	XP_002964599.1	25 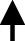	6 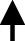	–	1	97	7.7/15	6.5/23
109	Quinonprotein alcohol dehydrogenase-like [*Medicago truncatula*]	gi|124360970|ABN08942.1	2.7 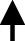	1.7 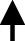	–	1	1	6.0/58	6.5/20
**CYTOSKELETON**									
39	Rec Name: Full = Actin	gi|5902734	2.4 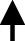	1.5 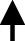	1.4 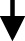	7	24	5.3/41	5.3/45
79	PREDICTED: WASH complex subunit strumpellin homolog [*Amborella trichopoda]*	XP_006844422.1	1.7 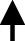	1.9 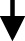	–	2	4	5.8/170	6.1/32
**EPIGENETIC CONTROL**									
49	Maturase K [*Cabomba caroliniana*]	gi|4106871	2.1 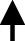	1.5 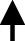	1.2 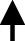	2	50	8.5/22	6.4/43
97	Related to JHD1-JmjC domain family histone demethylase specific for H3-K36 [*Piriformospora indica* DSM 11827]	CCA71072.1	1.8 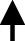	–	1.4 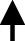	1	100	5.9/81	6.6/26
104	Related to JHD1-JmjC domain family histone demethylase specific for H3-K36 [*Piriformospora indica* DSM 11827]	CCA71072.1	2.1 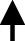	1.4 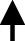	1.7 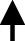	2	6	6.2/25	5.5/21
116	Maturase K [*Cabomba caroliniana*]	gi|4106871	1.4 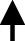	–	–	2	50	8.5/2	5.3/17
123	SET domain protein 35 [*Arabidopsis thaliana*]	NP_173998.2	3.0 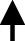	1.9 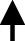	–	1	5.9	7.8/79	6.8/6
**STORAGE PROTEINS**									
1	Nutrient reservoir, putative [*Ricinus communis*]	XP_002533073.1	2.6 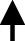	1.8 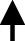	–	1	3.2	8.2/46	5.2/80
63	RmlC-like cupin [*Arabidopsis thaliana*]	NP_180436.1	1.9 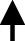	1.4 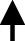	–	1	5.3	8.2/46	5.8/46
54	Cupin family protein [*Arabidopsis lyrata subsp. lyrata*]	XP_002881004.1	1.5 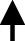	–	1.8 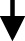	2	85	8.2/46	6.6/46
95	Cupin family protein [*Arabidopsis lyrata subsp*. lyrata]	XP_002881004.1	2.3 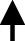	–	–	1	6	8.2/46	6.1/23
105	Glutelin type-A [*Medicago truncatula*]	gb|AET04449.1|	2.8 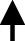	–	–	1	7	8.2/46	5.9/19
112	RmlC-like cupin *[Arabidopsis thaliana*]	gb|AAD24367.1|	1.6 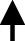	3.1 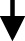	2.4 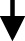	1	7	8.2/46	6.7/21
118	Glutelin type-A [*Medicago truncatula*]	XP_003605501.1	1.4 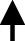	17.7 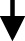	–	6	17.1	9.0/26	5.6/17
120	Hypothetical protein SELMODRAFT_159799 [*Selaginella moellendorffii*]	gi|302814437	2.8 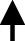	1.6 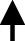	–	2	2	8/47	6.1/17
121	Cupin family protein [*Arabidopsis lyrata* subsp. lyrata]	XP_002881004.1	1.3 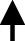	3.2 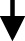	–	5	85	8.2/47	6.2/17
**MISCELLANEOUS PROTEIN**									
117	Hypothetical protein SELMODRAFT_428082 [*Selaginella moellendorffii*]	XP_002989542.1	2.0 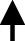	–	–	1	16.7	5.9/18	5.5/17
119	Hemolysin A [*Zea mays*]	NP_001152354.1	2.1 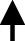	1.3 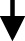	–	1	3.4	9.0/26	5.8/15
55	Aerobactin synthetase [*Grimontia hollisae*]	BAE16004.1	2.0 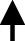	2.3 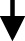	1.4	2	5	6.0/66	6.6/47
76	Hypothetical protein SELMODRAFT_407853 [*Selaginella moellendorffii*]	XP_002966726.1	1.92 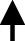	1.4 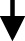	–	2	3	9.1/34	5.5/34
47	Predicted protein [*Physcomitrella patens* subsp. patens]	XP_001780580.1	3.3 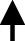	17.4 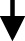	1.4 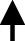	1	47	42/8.7	6.3/46

*Up regulated proteins are represented in red and denoted by upward arrow (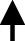) while down regulated proteins are represented in green with down arrow (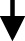);– denotes no significant change in comparison to control*.

Signal transduction plays a crucial role in triggering a cascade of defense and other metabolic events during stress. In roots several signaling proteins were found to be up-regulated e.g., short-chain dehydrogenase (SCDH spot 57; Table 1), protein phosphatase 2C family (spot 84; Table 1) and 14-3-3 protein (Spot 9; Table 1, Dataset S1 in Supplementary Information 2). LRR receptor-like serine/threonine-protein kinase (spot 106; Dataset S1 in Supplementary Information 2) was enhanced by 4 folds on DE which suggest its major role in dehydration tolerance because this protein almost disappeared on RI and came to its normal values on RII. Another protein which might be involved in ABA receptor and transportation activity was identified as ATP-binding cassette transporter subfamily C (spot 77; Table S1 in Supplementary Information 6).

Many proteins having anti-oxidative properties were found to be up-regulated on DE and RI (Table 1) including thioredoxin like protein (spot 113; Table 1), serine carboxypeptidase protein (spot 114), tau class glutathione S-transferease (spot 35), lactoylglutathione lyase (spot 81). Aldehyde dehydrogensae (ALDH) protein was found to be upregulated only during DE which is significant since ALDH is proposed to have a role in detoxification of lethal aldehydes.

Proteomic data also revealed some changes in cell wall proteins of roots e.g., phospholipase A1- gamma like protein (spot 45) and Glucan endo-1,3-alpha-glucosidase Agn1 (spot 60). Moreover, 5 protein spots identified as cupin (a storage protein) showed enhanced expression mostly during DE only. This protein has been reported to play a structural role in reinforcing the cell wall during stress.

Two proteins, up-regulated by 2 folds, belonged to category of protein synthesis. A significant increase in peptide chain release factor (spot 3; Table 1; Dataset S1 in Supplementary Information 2) and aminoacyl tR bNA synthetase (spot 96; Table 1) during DE and subsequent RI was found in roots of *Selaginella*. This shows that *S. bryopteris* roots were able to cope with dehydration by maintaining its protein synthesis machinery in stable state during dehydration/rehydration. It has been established that stress conditions affect cellular environments at least in part by disturbing protein folding. In roots, two spots of Hsp70 and HSP (spots 11 and 12; Table 1) were found to be up-regulated, on DE and on RI and RII respectively. These HSPs act as molecular chaperones for other proteins, thus preventing proteins from aggregating and denaturing.

It seems that cell division and root growth were not affected during water stress as two proteins, LAS1 protein (spot 20; Table 1) and UBX domain containing protein (spot 132) were found to be up-regulated on dehydration. In addition, a cytoskeleton protein, actin (spot 39; Table 1) was increased by more than two folds on DE and remained upregulated on R1 thereby providing much needed mechanical strength to roots.

In addition to oxidative stress, severe dehydration imposes a number of other stresses including metabolic and mechanical. Carbohydrate and energy metabolism play a crucial role in protective mechanisms. The two glycolytic enzymes (enolase; spots 29 and 32: Quinone protein alcohol dehydrogenase; spots 109 and 112, Table 1) increased in abundance during DE and rehydration. In addition, Glucose and ribitol dehydrogenase (spot 74; Table 1) exhibited 3 folds increase on DE and more than 2 folds on RI in roots of *S. bryopteris*. Two ATPase proteins (spots 52, 93) and a ATP binding protein (spot 108) were highly up-regulated on DE and latter remained increased by six folds on RI as well. This would have provided roots enough energy to cope up with the stress. Many proteins involved in nucleotide metabolism (e.g., nucleoside-triphosphatase/ nucleotide binding protein, pyrimidine specific ribonucleoside hydrolase rihA protein, nucleotidyltransferase family protein, DNA repair protein, RNA binding protein, pentatricopeptide repeat containing protein) were specifically upregulated during DE (Table 1) confirming their role in keeping the nucleotides in a proper conformation to ensure their activity during stress condition. Many epigenetic control related proteins like maturase and histone demethylases were also upregulated mostly during dehydration (Table 1).

Resurrection plants need more protection during rehydration because there are more chances of damage in cells during that process. In *Selaginella* most of the defense proteins (5 proteins) were up-regulated on first rehydration (RI) followed by carbohydrate and energy metabolism (5 proteins), signaling (3 proteins) and transcriptional control (2 proteins), while on second rehydration most of the proteins came to their normal values as compared to control (Figure 4).

### *S. bryopteris* frond proteomics

**Figure 3:**
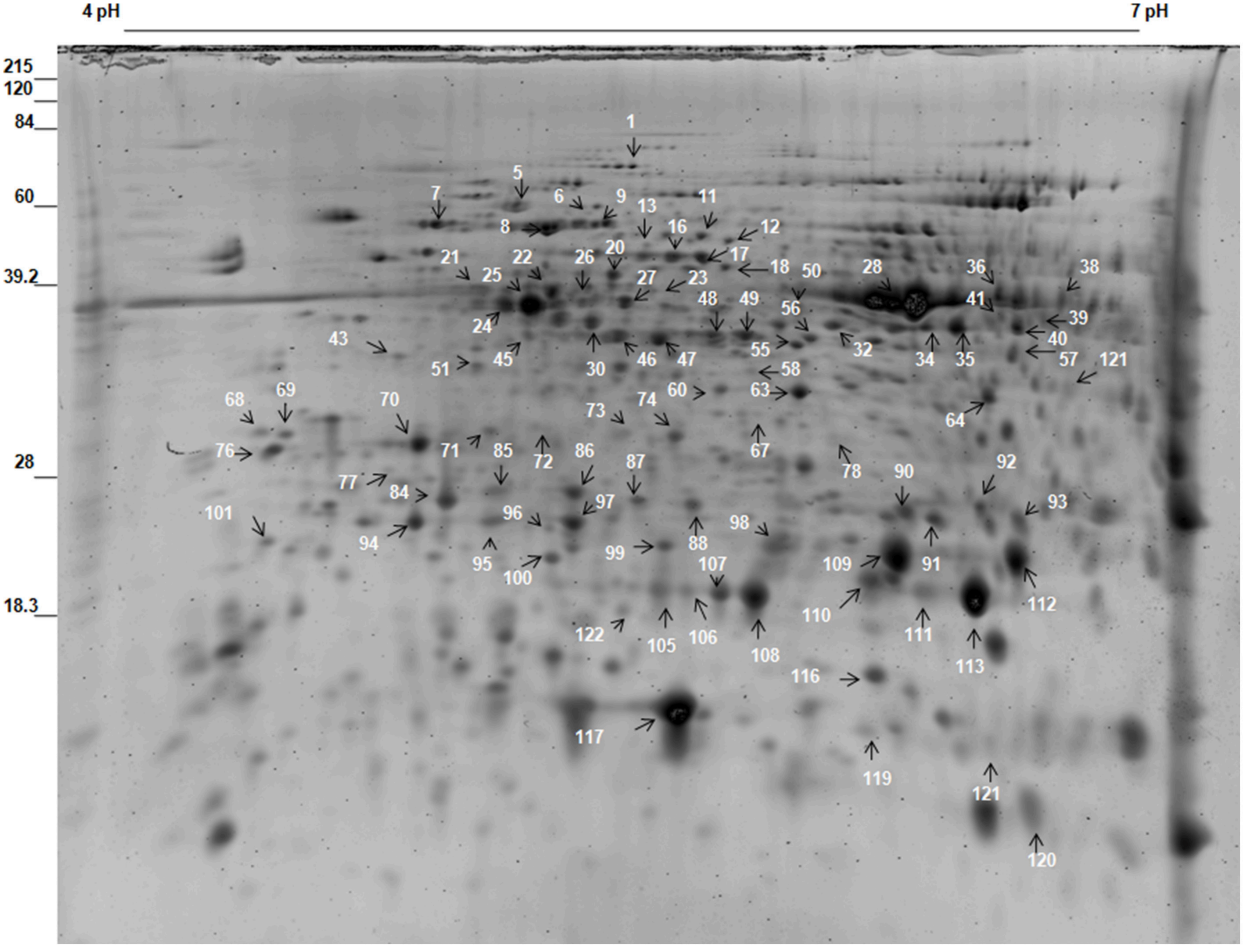
**Representative gel of ***S. bryopteris*** frond proteins**.

In total, more than 850 protein spots in fronds were reproducibly detected on sypro-ruby stained gels within each treatment. Out of these, 659 spots were matched to all the gels. The number of significantly differentially expressed proteins (*P* < 0.05) were found to be 121 out of which 87 spots were successfully identified by MALDI/TOF-TOF (Figure 3; Table S2 in Supplementary Information 6). Among the 87 identified proteins, different dehydration-responsive proteins covered various photosynthetic and metabolic pathways, including cell structure adaptation, photosynthesis protection, and different defense activities. The identified proteins were categorized among 9 broad functional categories. The proteins related to stress and defense (9 proteins; Table 2) protein metabolism (9), carbohydrate and energy metabolism (5) and photosynthesis (5) were highly up-regulated during dehydration. In contrast to roots, most of the proteins belonging above mentioned categories remained up-regulated on RI as well as on RII (Figure 5). In fronds, proteins most strongly affected under water deficiency included photosynthesis related proteins, stress and defense, heat shock proteins, and proteins related to carbohydrate and energy metabolism (Table 2).

**Figure 4:**
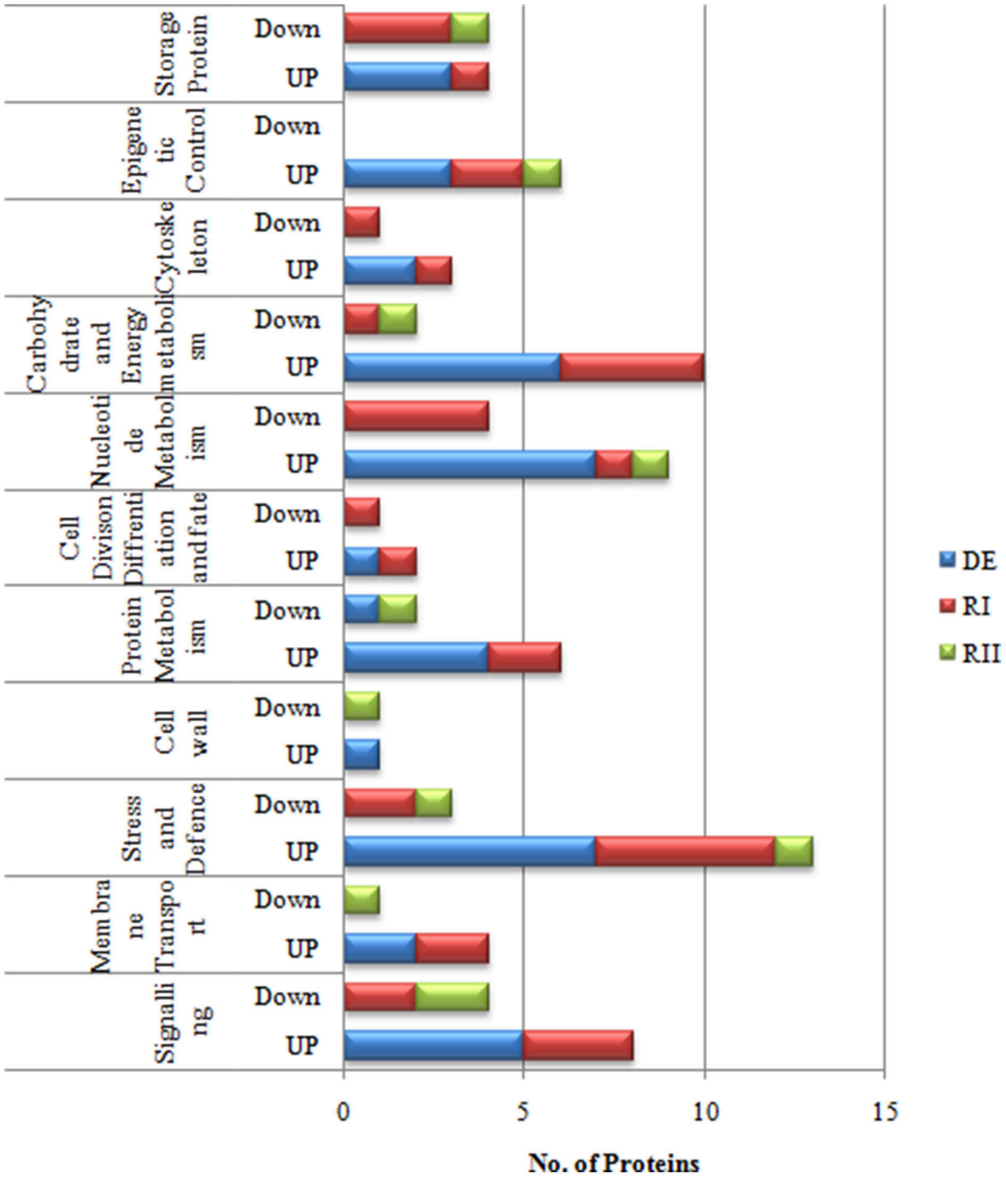
**Functional categorization of ***S. bryopteris*** root proteins**.

**Table 2:** **List of differentially expressed proteins in ***S. bryopteris*** frond during dehydration (DE) and Rehydration (RI and RII)**.

**Spot nos**.	**Protein**	**Accession no**.	**Differential Expression of proteins**			**Peptides matched**	**Sequence coverage (%)**	**Theort PI/MW**	**Observed pI/MW**
			**DE**	**RI**	**RII**				
**SIGNALING**									
77	14-3-3d protein [*Gossypium hirsutum*]	gi|164652940	2.6 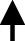	3.8 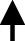	1.9 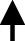	1	6	4.7/29	4.7/28
119	Nucleoside diphosphate kinase, NDPK=Nm23 protein homolog {N-terminal} {EC 2.7.4.6} [*Avena sativa*]	gi|619331	2.3 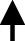	1.5 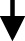	3.1 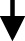	1	50	4.8/3	6.1/10
**STRESS AND DEFENSE**									
17	DAO-domain-containing protein [*Coccomyxa subellipsoidea* C-169]	XP_005648112.1	1.7 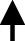	1.9 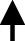	2.1 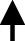	3	10.7	6.0/57.1	5.6/60
43	Late embryogenesis abundant protein Lea14-A, putative [*Ricinus communis*]	XP_002533345.1	–	1.5 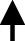	2.3 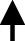	6	17.1	4.7/39	4.7/44
45	Putative peroxidase [*Cinnamomum micranthum* f. kanehirae]	gi|122726082	–	–	1.7 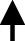	1	5	6.2/35	5.2/45
48	Monodehydroascorbate reductase [*Vitis vinifera*]	gi|146432261	2.2 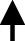	2.3 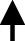	3.1 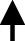	4	11	5.9/47	5.6/46
49	Plastidic glutamine synthetase precursor [*Brassica napus*]	gi|1934754	–	–	2.4 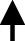	2	9	5.8/39	5.7/46
57	GDP-mannose 3,5-epimerase [*Arabidopsis thaliana*]	gi|15241945	1.6 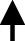	–	1.5 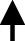	3	11	5.8/43	6.5/44
64	Dormancy related protein, putative [*Arabidopsis thaliana*]	gi|12322163	1.4 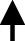	2.1 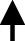	2.2 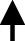	2	7	5.9/31	6.4/38
71	Desiccation-related protein, putative [*Arabidopsis thaliana*]	AAM65140.1	2.0 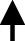	1.8 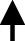	1.8 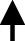	4	2	6.1/93	5.0/37
72	PREDICTED: desiccation-related protein PCC13-62-like [*Glycine max*]	XP_003546306.1	2.0 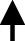	3.1 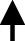	1.8 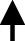	1	60	4.9/35	5.1/37
74	Lactoylglutathione lyase, putative [*Ricinus communis*]	gi|255546389	–	1.9 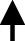	2.1 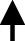	3	10	6.4/40	5.5/37
87	Ferritin, chloroplast precursor [*Physcomitrella patens* subsp. patens]	XP_001761934.1	2.1 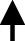	3.3 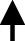	2.4 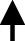	6	23.1	5.4/23	5.4/29
92	Ascorbate peroxidase [*Spinacia oleracea*]	gi|310587	1.7 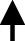	2.4 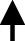	2.2 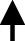	1	8	5.4/27	6.4/29
93	Glutathione S-transferase-like protein [*Solanum lycopersicum*]	NP_001234157.1	1.9 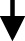	–	1.7 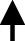	2	46	6.2/25	6.5/18
97	Tau class glutathione S-transferase [*Pinus tabuliformis*]	gb|AAV31760 AAT69969.1	1.8 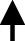	2.1 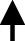	2.6 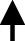	1	6	6.2/25	6.4/27
98	Dehydration responsive element binding protein [*Trifolium repens*]	ADD09598.1	1.8 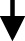	2.0 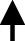	1.3 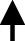	8	23	5.1/74	5.8/26
100	2-Cys-peroxiredoxin [*Riccia fluitans*]	gi|7339568	2.5 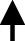	–	–	5	8	6.4/30	5.2/27
116	copper-zinc superoxide dismutase [*Nelumbo nucifera*]	gi|58615985	1.4 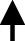	1.7 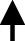	2.0 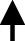	2	21	5.6/15	6.1/16
**PROTEIN METABOLISM**									
5	81kDa heat-shock protein [*Arabidopsis thaliana*]	gi|217855	2.0 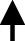	1.5 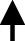	2.5 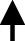	7	8	4.9/80	5.1/72
6	Chloroplast HSP70 [*Cucumis sativus*]	ABM92419.1	1.5 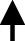	1.5 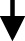	1.1 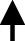	11	68.8	5.1/70	5.2/70
7	PREDICTED: stromal 70 kDa heat shock-related protein, chloroplastic-like [*Brachypodium distachyon*]	gi|357134135	1.8 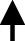	1.9 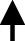	2.2 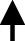	8	7	5.0/73	4.9/66
8	Stromal 70 kDa heat shock-related protein, HSP70 [*Triticum aestivum*]	gi|2827002	1.9 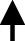	1.5 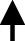	3.1	10	18	5.1/71	5.1/66
9	Luminal binding protein [*Pseudotsuga menziesii*]	gi|7635897	1.5 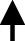	1.2 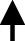	1.9 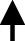	5	9	5.1/74	5.3/67
11	PREDICTED: heat shock 70 kDa protein, mitochondrial-like [*Glycine max*]	gi|356521247	1.3 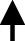	1.9 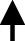	2.1 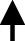	3	5	5.8/72	5.6/62
12	Membrane AAA-metalloprotease [*Chlamydomonas reinhardtii*]	gi|159478022	1.5 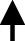	1.5 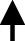	2.0 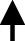	3	5	5.7/73	5.7/62
18	Chaperonin CPN60-like protein [Medicago truncatula]	XP_003591643.1	1.2 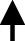	1.8 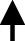	2.1 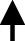	1	3.6	5.9/61	5.7/58
39	Serine/threonine protein kinase (Prp4), putative [*Aspergillus fumigatus* A1163]	EDP52695.1	1.7 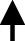	–	2.2 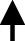	1	6.7	7.8/78	6.6/50
40	Transcription initiation factor TFIID, subunit TAF1 [*Physcomitrella patens* subsp. patens]	XP_001779301.1	1.6 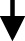	–	1.6 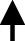	3	51	5.7/21	6.5/49
68	Cysteine protease [*Vicia sativa*]	gi|535473	–	2.2 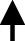	–	1	4	6.3/41	4.3/36
69	Cysteine protease [*Vicia sativa*]	gi|535473	1.4 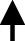	1.9 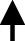	1.5 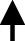	1	4	6.3/41	4.4/36
85	Ubiquitin thioesterase OTU1 [*Medicago truncatula*]	gi|357494501	2.7 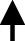	3.7 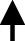	4.2 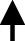	2	10	5.0/23	5.0/29
95	20S proteasome subunit beta-3	gi|17380183|O65084	–	2.0 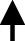	1.6 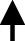	1	6	5.4/22	5.0/27
101	Cysteine protease [*Vicia sativa*]	gi|535473	3.5 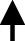	2.4 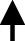	–	1	4	6.3/41	4.4/26
117	Ankyrin repeat-containing protein [*Arabidopsis thaliana*]	gi|15232175	1.3 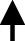	2.0 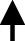	2.4 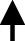	1	1	9.6/73	5.7/16
**CELL DIVISION DIFFERENTIATION AND FATE**									
1	Putative spindle disassembly related protein CDC48 [*Nicotiana tabacum*]	gi|98962497	1.6 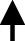	1.3 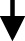	1.4 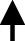	10	15%	5.1/90	5.4/80
67	Omega-amidase NIT2 [*Medicago truncatula*]	XP_003603190.1	4.9 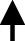	3.2 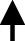	3.8 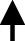	2	95	6.2/32	5.8/37
**NUCLEOTIDE METABOLISM**									
86	Pentatricopeptide repeat-containing protein [*Medicago truncatula*]	XP_003602631.1	2.0	2.0 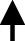	2.9 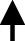	1	2.9	5.8/66	5.3/32
96	Pentatricopeptide repeat-containing protein, putative [*Ricinus communis*]	gi|255578711	3.0 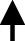	1.4 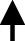	–	1	1	6.3/93	5.2/26
**CARBOHYDRATE AND ENERGY METABOLISM**									
16	ATP synthase CF1 alpha chain [*Selaginella moellendorffii*]	gi|255961300	1.8 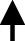	–	–	3	8%	5.3/54	5.5/58
20	ATP synthase CF1 alpha chain [*Selaginella moellendorffii*]	gi|255961300	2.0 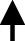	1.6 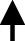	2.7 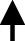	3	8%	5.3/54	5.3/54
22	Mitochondrial F1-ATPase beta subunit [*Dimocarpus longan]*	gi|269914683	1.8 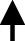	2.2 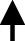	2.5	5	14%	6.1/59	5.3/53
25	ATP synthase subunit beta, mitochondrial; Flags: Precursor	gi|114421	–	–	1.7 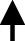	6	8	5.9/59	5.3/52
26	ATP synthase subunit beta, mitochondrial; Flags: Precursor	gi|114421	–	2.0 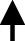	2.9 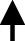	6	23	5.2/45	5.2/52
27	ATP synthase subunit beta, mitochondrial; Flags: Precursor	gi|114421	1.4 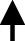	–	3.0 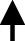	6	8	5.1/45	5.3/52
56	Phosphoglycerate kinase [*Oryza sativa* Indica Group]	gi|114386664	2.1 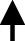	2.3 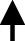	2.9 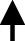	3	7	5.6/42	5.9/48
60	Fructose-bisphosphate aldolase	gi|357473565	1.9 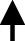	1.4 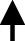	–	2	6	6.9/45	5.6/41
63	Fructose-bisphosphate aldolase	gi|357473565	1.7 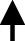	–	1.6 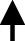	2	11	5.9/42	5.9/39
99	ATP binding protein, putative [*Ricinus communis*] gb|EEF29006	XP_002533375.1	1.6 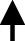	2.5 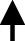	2.1 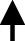	3	9	5.4/61	5.5/27
112	Quinonprotein alcohol dehydrogenase-like [*Medicago truncatula*]	gi|124360970|ABN08942.1	1.6 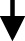	1.5 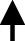	2.0 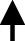	1	2	5.9/58	6.5/24
121	Glucose and ribitol dehydrogenase [*Medicago truncatula*]	gi|357441633|XP_003591094.1	3.5 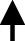	2.4 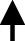	–	2	7	6.4/30	6.2/10
**CYTOSKELETON**									
21	Beta-tubulin [*Oryza sativa* Japonica Group]	gi|303842	0.8 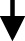	1.2 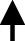	2.0 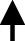	5	14%	4.7/50	5.0/55
46	Beta actin, partial [*Taxus cuspidata*]	gi|346683559	1.6 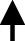	–	2.0 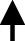	6	40	5.3/41	5.3/49
**EPIGENETIC CONTROL**									
90	Related to JHD1-JmjC domain family histone demethylase specific for H3-K36 [*Piriformospora indica* DSM 11827]	CCA71072.1	1.7 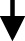	1.2 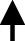	1.5 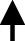	2	3	5.9/81	6.2/28
91	Trithorax-like protein, histone-lysine N-methyltransferase [*Physcomitrella patens* subsp. patens]	XP_001780587.1	1.7 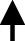	2.1 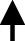	1.5 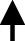	3	4	8.8/118	6.3/27
105	Maturase K [*Cabomba caroliniana*]	gi|4106871	1.5 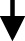	2.4 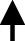	1.7 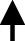	2	50	8.5/22	5.5/22
108	Maturase K *Cabomba caroliniana*]	gi|4106871	1.5 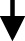	1.9 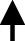	1.9 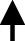	2	50	8.5/22	5.8/22
110	Maturase K [*Cabomba caroliniana*]	gi|4106871	1.9 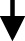	1.4 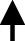	2.1 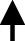	2	50	8.5/22	5.9/23
111	Maturase K [*Cabomba caroliniana*]	gi|4106871	3.3 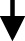	–	–	2	50	8.5/22	5.7/22
113	Maturase K [*Cabomba caroliniana*]	gi|4106871	1.4 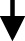	1.7 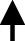	2.0 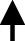	2	50	8.5/22	5.7/22
**STORAGE PROTEIN**									
41	Cupin family protein [*Arabidopsis lyrata subsp. lyrata*]	gi|297826243|XP_002881004.1	3.1 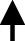	3.7 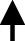	4.2 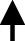	5	85	8.2/46	6.5/49
50	RmlC-like cupin [*Arabidopsis thaliana*]	NP_180436.1	–	1.7 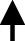	1.5 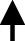	1	12.5	8.2/46	5.9/52
30	Cupin family protein [*Arabidopsis lyrata* subsp. lyrata]	gi|297826243|XP_002881004.1	1.8 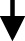	1.4 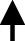	1.5 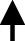	5	85	8.2/46	5.6/48
32	RecName: Full=Legumin A2;	gi|126161	–	1.8 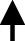	2.0 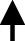	1	3	6.2/59	5.3/52
35	Cupin family protein [*Arabidopsis lyrata* subsp. lyrata]	gi|297826243|XP_002881004.1	1.5 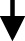	–	–	5	85	8.2/46	6.3/52
78	Beta-conglycinin, alpha chain;	gi|121281	1.9 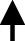	3.9 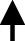	2.9 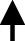	1	5.4	5.0/70.5	6.0/35
122	Beta-conglycinin, alpha chain;	gi|121281	1.7 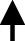	1.9 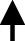	2.5 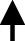	3	4	5.1/70	5.4/22
**PHOTOSYNTHESIS**									
55	Phosphoglycerate kinase, chloroplast, [*Musa acuminata*]	gi|102140037	2.2 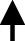	1.7 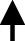	1.9 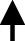	2	3%	8.7/50	5.9/46
23	Ribulose-1,5-bisphosphate carboxylase/oxygenase [*Selaginella digitata*]	CAC82458.1	–	–	1.6 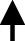	4	100%	6.1/47	5.5/54
28	Ribulose-1,5-bisphosphate carboxylase/oxygenase [*Selaginella digitata*]	gi|22859505	–	1.5 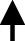	–	11	33%	6.2/47	6.2/52
34	Chloroplast elongation factor tub [*Nicotiana sylvestris*]	gi|297804102|XP_002869935.1	2.2 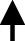	2.3 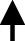	1.4 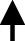	4	2%	5.5/72	6.2/48
36	Ribulose-1,5-bisphosphate carboxylase/oxygenase [*Selaginella digitata*]	gi|22859505	1.6 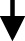	1.8 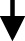	–	5	13	6.2/47	6.4/54
38	Ribulose-1,5-bisphosphate carboxylase/oxygenase [*Selaginella digitata*]	CAC82458.1	1.7 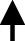	2.1 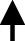	2.4 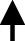	3	13.6	6.2/47	6.6/54
51	Sedoheptulose-1,7-bisphosphatase, chloroplast, putative [Ricinus communis]	gi|255579134	–	1.7 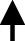	1.5 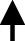	2	4	5.9/42	5.0/43
70	Oxygen-evolving enhancer protein 1, chloroplastic; *(Pisum sativum*)	gi|131384	1.7 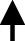	–	2.1 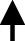	2	7	6.2/35	4.8/36
76	Chloroplast 29 kDa ribonucleoprotein [*Oryza sativa* Indica Group]		1.5 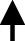	2.2 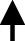	–	3	21	5.0/31	4.8/34
84	Chlorophyll a/b binding protein of LHCII type I [*Wolffia australiana*]	gi|374412428	1.7 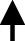	1.9 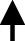	–	4	13	5.4/28	4.9/28
94	Light-harvesting chlorophyll a/b-binding protein of photosystem II [*Cryptomeria japonica*]	gi|3417451	2.3 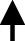	1.8 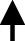	1.6 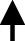	4	10	5.6/28.6	4.9/28
47	Rubisco activase [*Medicago sativa*]	gi|23320705	–	–	1.7 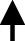	3	25	5.6/30	5.5/44
107	Oxygen-evolving enhancer protein 2[*Arabidopsis lyrata* subsp. lyrata]	XP_002881132.1	1.6 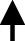	1.7 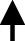	–	6	17	5.9/23	5.7/23

*Up regulated proteins are represented in red and denoted by upward arrow (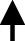) while down regulated proteins are represented in green with down arrow (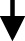);– denotes no significant change in comparison to control*.

**Figure 5:**
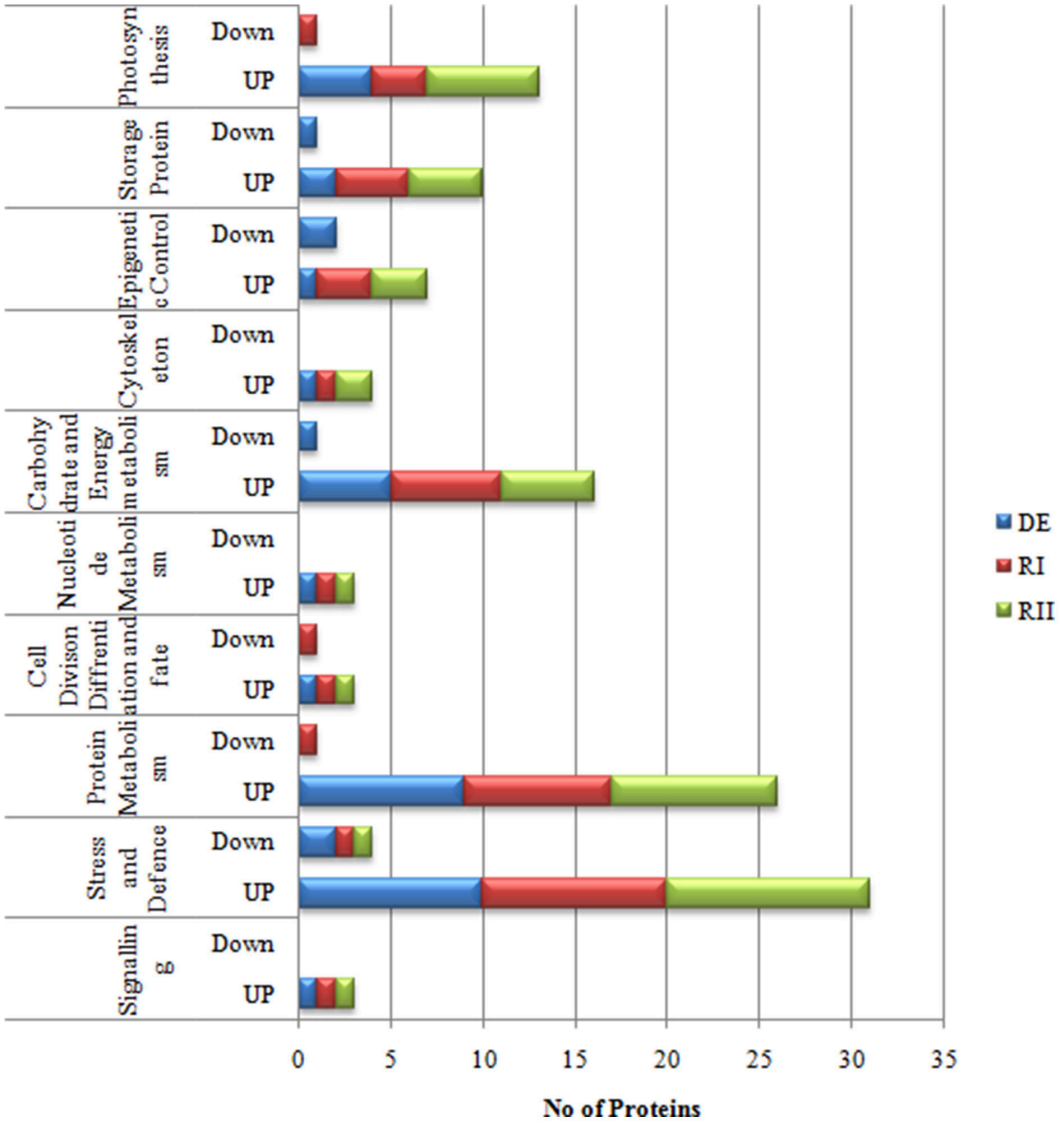
**Functional categorization of ***S. bryopteris*** frond proteins**.

Photosynthesis is highly sensitive to periods of water deficit. The primary enzyme involved in carbon fixation is ribulose-1, 5-bisphosphate carboxylase/oxygenase (Rubisco). In the present experiment 3 Rubisco subunits remained stable or increased on DE (spots 23, 28, 38; Table 2) except one which was decreased (spot 36). Rubisco activase, which restores the catalytic activity of Rubisco, was found to be increased on second rehydration (spot 47, Table 2). Interestingly several other enzymes involved in carbon fixation increased in abundance or remained unchanged during dehydration in *Selaginella* including chloroplastic phosphoglycerate kinase, sedoheptulose 1,7-bisphosphatase, fructose bisphospahte aldolase (Table 2; Dataset S2 in Supplementary Information 2). The accumulation of these enzymes suggests that a partial Calvin cycle may be required for the establishment of dehydration tolerance in *Selaginella*. As *Selaginella* is a homoiochlorophyllous plant, its photosynthetic structure needs to be protected. It was not surprising that many different proteins were involved in maintenance of chloroplast stability in *Selaginella* during dehydration e.g., chl a/b binding protein (spots 84, 94), oxygen evolving enhancer protein (spots 70, 107), chloroplast EF-Tu (spot 34). This further indicated that the integrity of thylakoid membranes was maintained during dehydration and subsequent rehydration.

There was massive induction of stress and defense related proteins in response to dehydration and rehydration. Many proteins showed enhanced expression at dehydration and also at both the rehydrations e.g., SOD, APX and DHAR, GST, desiccation and dormancy related proteins, a ferritin although a LEA, DREB, lactoylglutathione lyase proteins were induced only on RI or RII, indicating their roles during rehydration. Significantly, level of GDP mannose 3 5 epimerase (spot 57) was found to be up-regulated during DE and RII. This enzyme represents the first step in the de novo synthesis of ascorbate. Besides a number of heat shock proteins, mainly HSP70, a chaperonin like protein, luminal binding proteins were also upregulated during dehydration. Additionally proteins related to ubiquitin/proteasome mediated protein degradation and some cysteine proteases were also up-regulated during dehydration, highlighting possible involvement of these proteins in stress response and substantiating the notion that cleavages of specific target proteins contribute to the events that accompany dehydration and subsequent rehydration.

Several of the most abundant proteins in drought stressed samples were related to energy metabolism. Two proteins were identified as ATP synthase CF1 alpha chain (spots 16, 20; Table 2) and 4 were identified as mitochondrial ATP synthase beta subunits (spots 22, 25, 26, 27). Expression of all the six proteins increased throughout the whole experiment but alpha subunit played major role during DE stage while beta subunits dominated in rehydration cycles (RI and RII). Enhanced expression of ATP synthase beta subunits which were highly up-regulated during rehydration would have lead to an increased supply of ATP for various cellular processes needed for damage repair during rehydration.

Two signaling related proteins showed differential expression pattern. While 14-3-3d protein remained upregulated throughout the experiment, more than two folds increase in nucleoside diphosphate kinase (spot 119; Table 2) was found during dehydration and a decrease on both rehydrations. NDPKs play significant roles in hormone responses, heat stress, drought stress, mitogen-activated protein kinase (MAPK)-mediated H_2_O_2_ signaling, growth, and development.

About five fold increase in omega amidase NIT2 protein (spot 67; Table 2) was observed during DE and it remained over expressed during RI and RII. This protein is reported to play role in nitrogen cycle. In humans, though, role of omega-amidase is reported to remove potentially toxic intermediates by converting alpha-ketoglutaramate and alpha-ketosuccinamate to biologically useful alpha-ketoglutarate and oxaloacetate, respectively. Such a high expression of this protein in *Selaginella* fronds assumes significance and needs further investigation.

Two spots of pentatricopeptide repeat containing proteins (PPR, spots 86, 96; Table 2) were found to be significantly up-regulated throughout the experiment. While many epigenetic control related proteins were differentially regulated. Four spots of maturase proteins (spots 105, 108, 110, 111) were down regulated during DE but were upregulated on rehydrations. While a trithorax like protein (spot 91) remained up-regulated throughout the experiment. Many storage proteins like cupin family protein (spots 30, 35, 41, 50), a legumin (spot 32) were mostly down regulated during dehydration but were expressed more at RI and RII (Table 2).

### Biplot analysis

We performed the principal component analysis (PCA) of the relative abundance data for 59 proteins in roots and 88 proteins in fronds. Principal component (PC) 1 explained about 92% of the variance in the dataset while 4% was contributed by PC2 in roots (Figure 7A). Similarly 94% variance was exhibited by PC1 in fronds. Thus, the major variance in PC1 clearly distinguished the two treatment observation in roots and fronds, respectively. The norms of reaction plots for PC1 and PC 2 reiterate this interpretation that these axes together reveal significant interaction between protein expression and imposed treatment (Figure 7B; Supplementary Information 5). Distribution of proteins along the two components (PC1 and PC2) clearly indicates their variance according to their treatment e.g., in roots, all the proteins are directed toward the DE treatments that is completely in the another plot as compared to Con, R1 and R2. On the other hand, in fronds most of proteins were separated toward rehydrations (Figure 7B; Supplementary Information 5).

## Discussion

Resurrection plants have evolved the ability to withstand cellular dehydration in their vegetative tissues. Water deficit induces many morphological changes in dehydration-tolerant plants, the most obvious of which is leaf folding (Le and McQueen-Mason, 2006; Nar et al., 2009). The fronds of *S. bryopteris*, which are fully expanded when watered, progressively curl inward during drying and become tightly folded, so that only the abaxial surfaces of the fronds are exposed to the sun (Pandey et al., 2010). Leaf folding limits photo-oxidative damage from light stress, decreases the transpiring area and is thus an important morphological adaptation for surviving dehydration (Brighigna et al., 2002; Nar et al., 2009). This process is reversible after rehydration.

**Figure 6:**
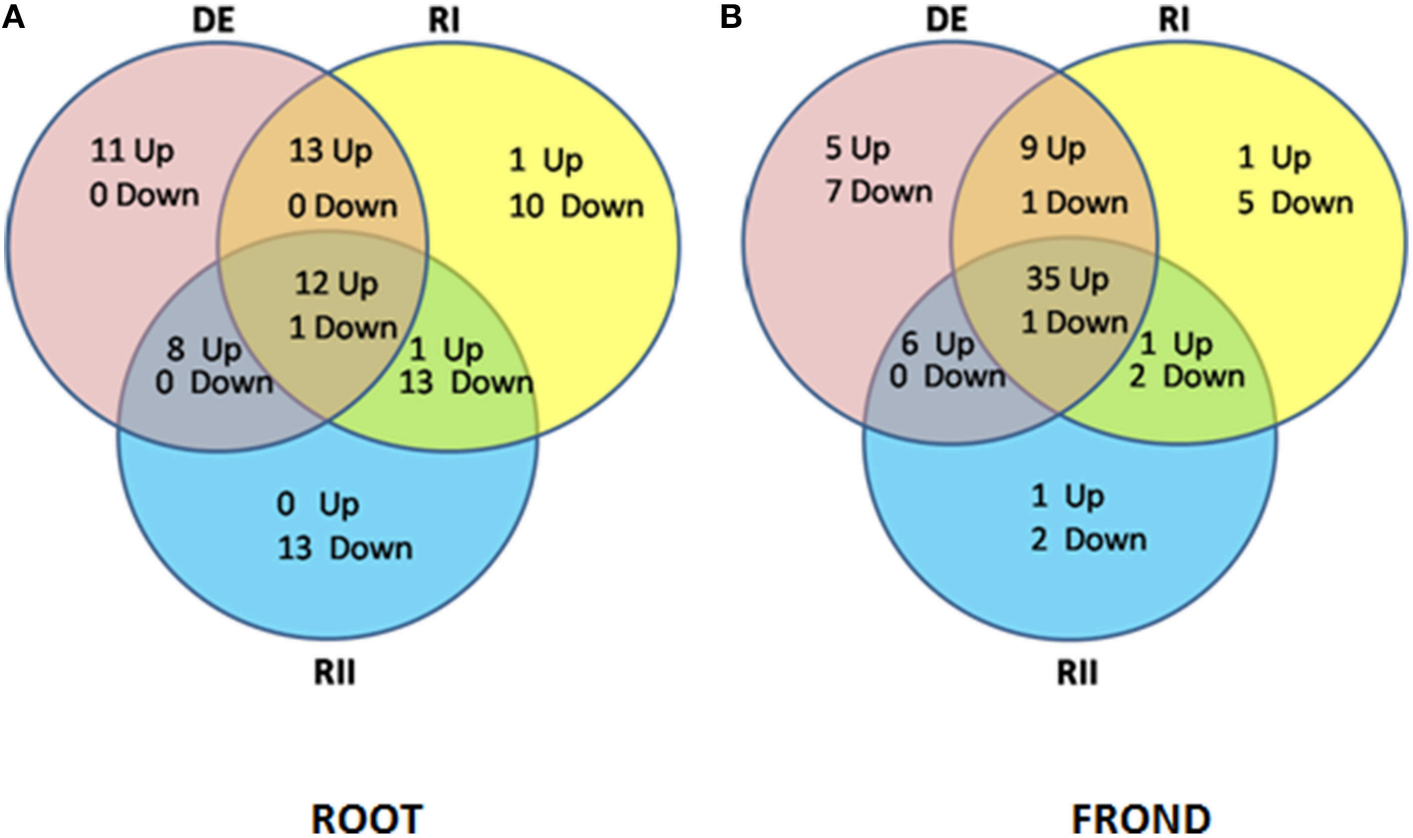
**Venn diagram analysis illustrating the (i) up regulated proteins, and (ii) down regulated proteins in (A) roots and (B) fronds during dehydration (DE) stress and rehydration (RI and RII) in ***S. bryopteris*****.

In the present study, proteomic work was conducted to determine the type of dehydration tolerance in *S. bryopteris*. The plants survived 7 days without watering and recovered to a normal condition 24 h after rewatering. This is the first study of the root system of *S. bryopteris* under dehydrated as well as rehydrated conditions. In *S. bryopteris* roots majority of the proteins were up-regulated during DE and RI. While in case of fronds majority of the proteins were up-regulated on DE and RI and RII as well (Figures 6A,B). This indicated that response to dehydration stress in roots was inductive while in fronds it was constitutive.

**Figure 7:**
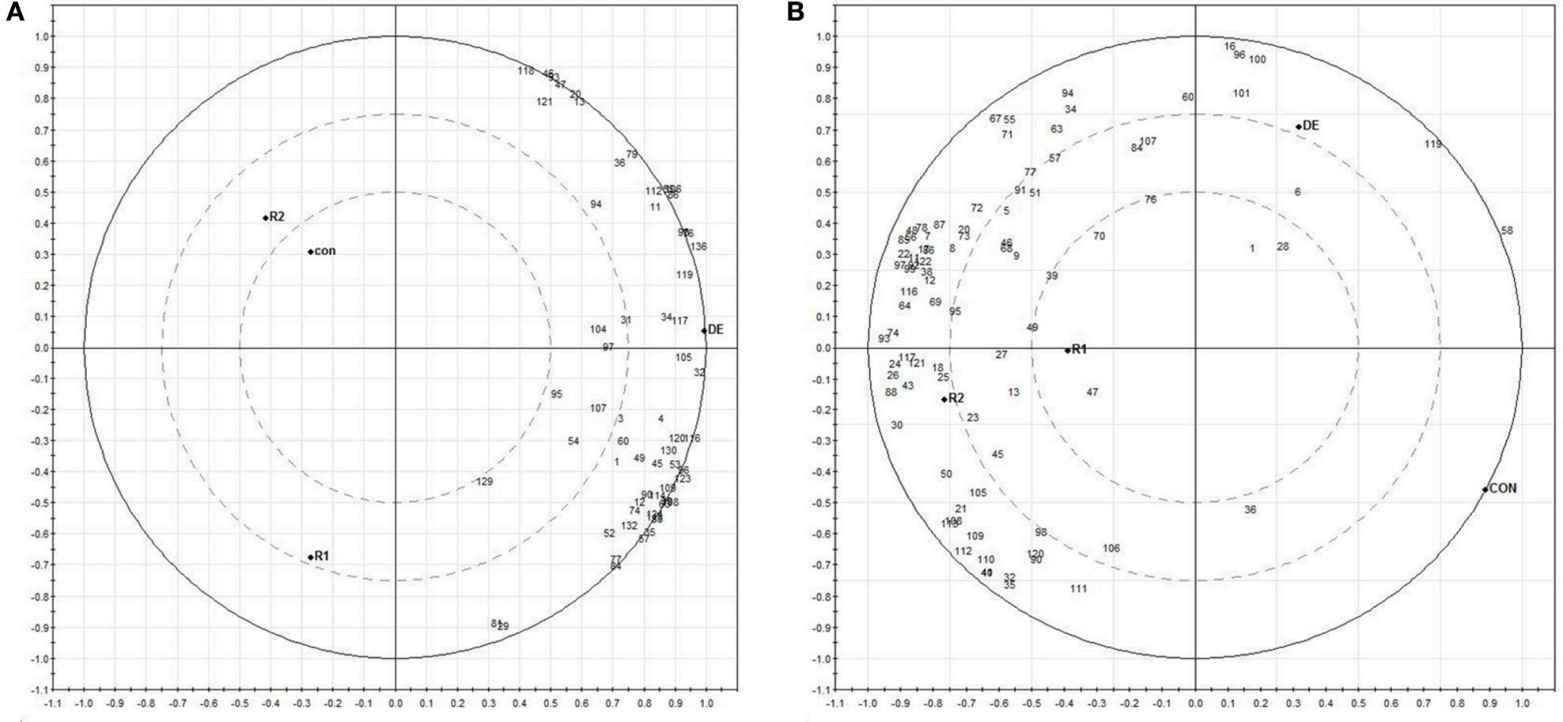
**Biplots based on PCA results from differentially expressed proteins of roots (A) and fronds (B) during dehydration (DE) and rehydration**.

The apparent lack of cell damage and severe oxidative stress shows that *S. bryopteris* is indeed a genuine resurrection species. Plant metabolism was finely coordinated with the induction of strong stress defense both in roots and fronds which provided protection against water deficiency. A stable or induced photosynthesis related proteins helped plant recover quickly upon rehydration.

### Signal transduction

Signal transduction plays a crucial role in triggering a cascade of defense and other metabolic events. In the present study we found increased expression of several signaling related proteins during dehydration in both roots and fronds and most of the proteins were found to be up-regulated, more so in roots. Protein phosphorylation and dephosphorylation are essential signaling events leading to acquisition of drought tolerance. Two most drought and RI upregulated proteins in roots were short chain dehydrogenase and phosphatase 2C. Both proteins are involved in biosynthesis and signaling of ABA, respectively as evidenced in *Arabidopsis* (Endo et al., 2008). Involvement of ABA in the systemic drought response is now well established (Christmann et al., 2005). A LRR receptor-like serine/threonine-protein kinase (spot 106; Dataset S1 in Supplementary Information 2) was enhanced by 4 folds during DE which suggest its major role during dehydration because this protein almost disappeared on rehydration (RI and RII) in *S. bryopteris* roots. This protein is increased by ABA mediated signaling pathway during drought stress and over-expression of GbRLK (*Gossypium barbadense* Receptor like kinases) has been shown to improve the salt and drought tolerance in transgenic *Arabidopsis* (Zhao et al., 2013). A protein with remarkable ABA receptor properties, ATP-binding cassette transporter subfamily C (spot 77; Table S1 in Supplementary Information 6), was enhanced on DE and RI in *S. bryopteris* roots. Recently, two plasma membrane ATP-binding cassette (ABC) transporters have been identified in *Arabidopsis*, giving further insight into the influx/efflux mechanism of ABA and providing information on how ABA is transported from cell to cell in plants (Kang et al., 2010; Kuromori et al., 2010). This protein was also reported to be enhanced under dehydration in *Boea hygrometrica* (Jiang et al., 2007). Our results show that *Selaginella* roots posses a highly efficient signaling network to cope with water deficiency.

In both roots and fronds, 14-3-3 proteins were upregulated during dehydration. These proteins are the indispensible regulators in plant growth and development, and also play important roles in response to abiotic stress (Deeba et al., 2012). On rehydration most of the signaling proteins showed variable responses.

Growth cessation is normally observed in plants experiencing water stress, along with alterations in cell cycle and cell wall related proteins. But in our study we found enhanced expression of cell growth related proteins like LAS1 and UBX domain proteins in roots. A root phospholipase A1-gamma like protein (spot 45) was increased during dehydration. This enzyme catalyses hydrolysis of phospholipids forming lysolipids and fatty acids. In *Sporobolus stapfianus*, accumulation of lysolipids suggests the scope for minimal damage to lipid membranes during dehydration (Oliver et al., 2011). These alterations in unsaturated fatty acid concentrations are supposed to contribute to membrane fluidity to tolerate dehydration stress (Upchurch, 2008).

### Photosynthesis of *S. bryopteris* and role of heat shock proteins and antioxidant defense

The strategy of retaining chlorophyll and photosynthetic machinery is potentially dangerous as excessive ROS may be produced upon illumination of the remaining chlorophyll. That is why resurrection plants like *S. bryopteris* have evolved various strategies to cope with oxidative stress. One obvious morphological adaptation is leaf folding which minimizes frond surface area. The increased abundance of some of the enzymes which also have a role in glycolysis may indicate a shift between autotrophy and heterotrophy during dehydration (Griffiths et al., 2014). In our study, protection to photosynthetic machinery during dehydration as well as rehydration was provided by various proteins like oxygen evolving enhancer protein (OEE), chl a/b binding protein, and chloroplast elongation factor. OEE stabilizes the catalytic Mn cluster of photosystem II and regulates the turnover of the D1 reaction center protein (Lundin et al., 2007). Merewitz et al. (2011) reported increased expression of chloroplast EF-Tu in drought tolerant transgenic creeping bentgrass overexpressing an *ipt* gene for cytokinin biosynthesis. In addition several heat shock proteins were highly induced by DE. Two chloroplastic HSP70s, 2 stromal HSPs, one mitochondrial and a luminal binding protein were upregulated throughout the experiment. Members of the Hsp70 chaperone superfamily play a central role in facilitating the folding, unfolding, and transport of a wide range of proteins (Wang et al., 2004). In addition, these chaperones appear to be involved in the recognition and turnover of misfolded destabilized proteins thereby ensuring a suitable environment for cellular function (Hartl and Hayer-Hartl, 2002). Members of the Hsp70 family have been implicated in the targeted delivery of proteins to specific cellular domains (Tsai et al., 2000) other than to organelles like the peroxisome, mitochondrion, chloroplast, and endoplasmic reticulum (Hendershot, 2000). These proteins are also involved in protein import and translocation processes, and in facilitating the proteolytic degradation of unstable proteins by targeting the proteins to lysosomes or proteasomes (Hartl, 1996). Moreover, recent studies suggest that HSP70 acts as a key regulator in the formation of anisotropic interdigitation i.e., interlocking marginal lobes (IMLs) involving the cell wall–cell membrane–cortical actin continuum, in drought-tolerant plants (*Erianthus arundinaceus* and HSP70 overexpressing transgenic sugarcane) under moisture stress (Augustine et al., 2015).

These findings indicate that the preservation of photosynthetic structure in *S. bryopteris* and other important proteins during dehydration in nature is facilitated by activation of large number of stress protective proteins.

Enhanced expression of many proteins related to protein degradation and turnover (AAA-metalloprotease, cysteine proteases, 20S proteasome, ubiquitin thioesterase), both during dehydration and rehydration suggests that these proteins are important for survival of *Selaginella* fronds, since these proteins were expressed only in fronds. Earlier study on *S. bryopteris* also showed the possible involvement of proteins involved in transport, targeting and degradation were more expressed during dehydration (Deeba et al., 2009). Presence of cysteine proteases in *Selaginella* fronds is little perplexing as these are found in tissues undergoing oxidative stress-mediated programmed cell death (Solomon et al., 1999). It is therefore possible that severe dehydration triggers their expression to initiate cellular recycling programme. Transcripts for several types of cysteine proteases and 2 protein spots were also found in dehydrating *Craterostigma plantagineum* leaves (Rodriguez et al., 2010). Though cell protection mechanisms are considered to play important role in dehydration tolerance, our study indicates that role of repair mechanisms, as represented by these proteins, may be more than supplemental. This ability of *S. bryopteris* to accumulate these proteins during dehydration and rehydration suggests strategic role in rapid recovery from dehydration.

Recovery of a resurrection plant correlates with its capacity to establish a number of antioxidant protective mechanisms during dehydration and to maintain these systems upon rehydration (Kranner et al., 2002). There were six defense related proteins in roots which were found to be up-regulated on dehydration and RI e.g., aldehyde dehydrogenase, thioredoxin, serine carboxypeptidase, leucine rich repeat family protein, tau class glutathione S-transferase. However, in fronds more number of defense related protein were found to be up-regulated during dehydration. Out of 12 defense proteins in fronds, 2-Cys-peroxiredoxin, MDHAR were up-regulated by more than two folds on dehydration and remained upregulated on RI as well. While SOD, APX, DHAR, GST, 2 desiccation related proteins, DREB remained over-expressed throughout the experiment. Similar results have been reported by Wang and co-workers (Wang et al., 2009) in *Physcomitrella patens*. Ferritin protein (induced during DE and RI in fronds) is highly conserved and plays a critical role in iron storage and homeostasis (Murgiaa et al., 2001). The storage function of ferritins has been associated with a cytoprotective antioxidant effect against lethal hydroxyl radicals. Additionally, lactoylglutathione lyase protein was found to be increased dehydration and rehydration both roots and fronds. Lactoylglutathione lyase is one of the enzymes of glyoxalase system that removes cytotoxic methyl glyoxals. Sun et al. (2010) found increased expression of 2 genes encoding for lactoylglutathione lyase in salt-tolerant wild tomato species. Thus, our results strengthen the notion that *S. bryopteris* possess potent antioxidant protein network. In *Craterostigma wilmsii* and *Xerophyta viscosa*, increased expression of SOD, APX and GR genes during dehydration or rehydration has been reported (Ingram and Bartels, 1996; Sherwin and Farrant, 1998). Surprisingly, LEA protein (spot 43) played its part only during rehydration in fronds. In rehydrating *T. ruralis* gametophytes, LEA proteins function in stabilizing membranes, or perhaps in the transport of lipids for reconstitution of damaged membranes (Oliver et al., 2005). On the other hand, increased expression of LEA was observed during dehydration stress in *S. tamariscina* (Wang et al., 2010). The results are consistent with the hypothesis that plants allocate more carbon to anti-stress mechanisms under drought stress.

### Carbohydrate and energy metabolism

In addition to mechanical and oxidative stress, severe drought imposes a number of other stresses, most notably metabolic. Carbohydrate and energy metabolism played a central role in protective mechanisms in our study, as 8 proteins in roots (Table 1) and 12 proteins in fronds (Table 2) were up-regulated during dehydration. In roots, 2 protein spots of enolase were overexpressed during dehydration and one remained upregulated on RI and RII as well. Enolase catalyses the conversion of 2-phospho- glycerate to phosphoenolpyruvate during glycolysis, and catalyses the reverse reaction in gluconeogenesis. An increase in flux through the gluconeogenic pathway during drying would provide an increased pool of hexose phosphate substrates required for both sucrose and sorbitol synthesis. Carbohydrate metabolism is modulated in resurrection plants during drying, particularly toward the synthesis of sucrose (Bianchi et al., 1991; Whittaker et al., 2001), and possibly toward the synthesis of compatible solutes such as sorbitol (Mundree et al., 2000) or ribitol (Yobi et al., 2013). Three more glycolytic enzymes, phosphoglycerate kinase, glucose and ribitol dehydrogenase and quinonprotein alcohol dehydrogenase also showed differential expression during dehydration and rehydration. Glucose and ribitol dehydrogenase has been implicated in an alternative carbohydrate metabolism during embryogenesis (Alexander et al., 1994). Phosphoglycerate kinase also played a key role in glycolytic pathway fulfiling the energy requirement during dehydration in *S. bryopetris* fronds. Cui et al. (2012) also found increased abundance of 2 phosphoglycerate kinase proteins in *Physcomitrella patens* both during dehydration and rehydration.

In roots and fronds, many proteins related to ATP synthesis process were significantly up-regulated during dehydration and rehydration. Many members of ATP synthase family including ATP synthase CF1 alpha chain, mitochondrial ATP synthase beta subunits, mitochondrial F1-ATPase beta subunit and ATP binding proteins were enhanced to varying degrees in both roots and fronds during dehydration and rehydration. Whereas alpha subunit played major role during DE, beta subunits dominated in rehydration cycles (RI and RII) in *S. bryopteris* fronds. Since mitochondrial ATP synthase beta subunit is known to be involved in ATP hydrolysis and ATP biosynthesis coupled to proton transport, their increase indicate enhanced demand for ATP during rehydration. Wang et al. (2010) also found increased abundance of seven ATP synthase proteins in resurrection plant *Selaginella tamariscina* under desiccation stress. The authors attributed this abundance as the fundamental requirement of desiccation tolerance. Because activation of ATP synthase will decrease proton gradient across the thylakoid membrane and enhance energy transduction between PSII and PSI (Braun et al., 1991) the over expression of CF1-alpha isoform may indicate a regulatory pathway to prevent from over protonation of thylakoid lumen and damage of photosynthetic apparatus under drought stress. Similar results were also found by Macarisin (Macarisin et al., 2009) in crab apple (*Malus pumila*). ATP binding proteins have important roles in membrane transport, cellular motility and regulation of various metabolic processes. Our results indicate that there was an increase in carbon metabolism and energy production to cope up with stress and helping in recovery.

### Epigenetic control and storage proteins

Epigenetic modification is defined as changes in gene activity without changes in the original DNA sequence. These changes can be transferred to cell's progeny during mitosis or meiosis (Chen et al., 2010). Furthermore, these changes can be mediated at several independent levels, including DNA methylation, histone post-translational modifications etc. (Chinnusamy and Zhu, 2009). In roots three proteins related to histone demethylase were found to be regulated during DE. While in fronds, a histone demethylase and a methyltarnsferase were found to be drought responsive. Plant SET-domain family member proteins play decisive functions in various processes including cell fate determination, leaf morphogenesis, parental imprinting and seed development (Liu et al., 2010; Berr et al., 2011). The Tri-methylation of histone H3 at lysine 4 (H3K4me3) in Arabidopsis by TRX-like factor ATX1, is shown to participate in dehydration stress signaling in both ABA-dependent and ABA-independent pathways (Ding et al., 2011). A remarkable increase was also found in transcription of HvTX1 encoding a TRX-like H3K4 methyltransferase in barley upon drought treatment (Shvarts Iu et al., 2010). These studies indicated that plant TRX-like factors play a crucial role in plant response to environmental stresses. Moreover, rice JMJ703 was observed as a histone lysine demethylase that specifically demethlases all three forms of H3K4me in rice (Chen et al., 2013; Cui et al., 2013). Loss-of-function mutation of JMJ703 affects stem elongation and plant growth and leads to mis-regulation of the activities of transposons in rice (Chen et al., 2013; Cui et al., 2013). These studies suggest that JMJ proteins play essential roles in plant development and gene silencing.

Moreover, many maturae K proteins were differentially regulated in roots and fronds. Their identification as drought responsive protein suggests involvement of chromatin remodeling in the response of *Selaginella* roots and fronds to drought stress. Chromatin remodeling is an important mechanism in transcriptional reprogramming in responses to various stresses (Claeys and Inze, 2013). Thus, our results show that stress memory appears to be inherited through epigenetic changes, giving *Selaginella* an adaptive advantage. The process of dehydration acclimation is associated with mobilization of energy reserves and an enhanced need for components for de novo biosynthesis of proteins under dehydration stress. We found increased abundance of cupin proteins in both roots and fronds during dehydration. Cupin superfamily represents an important source of energy and as well as amino acids, which can be utilized for a de novo biosynthesis of proteins under dehydration stress. These proteins have also been reported to play a structural role in reinforcing the cell wall during pathogen attack (Schweizer et al., 1999). Expression of such a high number of cupin proteins both in roots and fronds showed that these proteins played a vital role during dehydration in *S. bryopteris*.

### Nucleotide metabolism

Roots of *S. bryopteris* proteins exhibited more changes under this category than fronds. There were 6 proteins (7 spots; Table 1) which were upregulated mostly during DE. The pentatricopeptide repeat (PPR) is a family of putative RNA binding proteins known to mediate specific RNA processing events, including RNA editing, transcript processing, and translation initiation. PPRs are thus capable of specific binding to both protein and RNA molecules (Deeba et al., 2012). Liu et al. (2008) reported up-regulation of PPR protein in desiccating *S. tamariscina*. We found another RNA binding and a DNA repair protein high in abundance. A nucleoside-triphosphatase/ nucleotide binding protein were crucial in maintaining protein synthesis turnover during water stress conditions. There were two fold increases in the expression of pyrimidine specific ribonucleoside hydrolase rihA protein in roots during DE (spot 94 in Table 1). Nucleoside degradation and salvage are important metabolic pathways but hardly understood in plants. Petersen and Moller (2001) reported in *Escherichia coli* that both *rihA* and *rihC* were subjected to catabolite repression might suggest a role for these genes in the provision of ribose for utilization as a carbon source. Increased expression by more than two folds of nucleotidyltransferase family protein (spot 130; Table 1) on DE in roots of *Selaginella* suggests its role in tRNA synthesis for active protein synthesis. This study is consistent with the other protein involved in protein synthesis. Our results clearly show that *Selaginella* roots were able to protect its nucleotide machinery during dehydration stress.

## Conclusion

Roots and fronds of *S. bryopteris* followed slightly different strategies to cope with water stress as reflected by protein levels. Most striking response was shown by roots as, barring one, all the proteins showed higher abundance during dehydration and on rehydration most of them either came to control level or were down regulated. This clearly shows that higher abundance of proteins in roots of *Selaginella* was inductive due to dehydration. Understandably, fronds showed higher number of protein expression changes as compared to roots. There was an overlap of protein abundances induced during dehydration and rehydration in both roots and fronds although number of proteins and their expression levels varied. High level of overlapping pointed to common mechanisms that allowed plant adaptation to stress and helped in recovery. For example in both the organs there was an increased abundance of key enzymes of energy metabolism to increase ATP production: in roots it was more during dehydration while in fronds it was more at rehydration. Enhanced levels of these primary metabolism related proteins thus indicate that adequate energy supply is a pre-requisite for these organs to deal with water deficit. Photosynthesis was inhibited but photosynthetic apparatus was protected by increased abundance of several proteins. This necessitated expression of more ROS scavenging proteins in fronds (17) than in roots (7). Moreover, proteins involved in proteolysis, protein folding, and storage were found to be high in abundance that indicate their probable involvement in excluding damage induced non-active proteins. One of the examples of these categories include heat shock proteins that function as a molecular chaperone in variety of cellular processes such as prevention of protein aggregation, translocation of nascent chains across membranes, assembly, or disassembly of multimeric protein complexes, and targeting proteins for lysosomal or proteasomal degradation. The other possible novel regulator in dehydration tolerance may be represented in epigenetic regulation. Recent studies have linked epigenetic modifications with drought tolerance which could provide within generation and trans-generational stress memory. Other proteins with unknown functions or no sequence homology are a potential source for gene discovery involved in drought tolerance.

**Figure 8:**
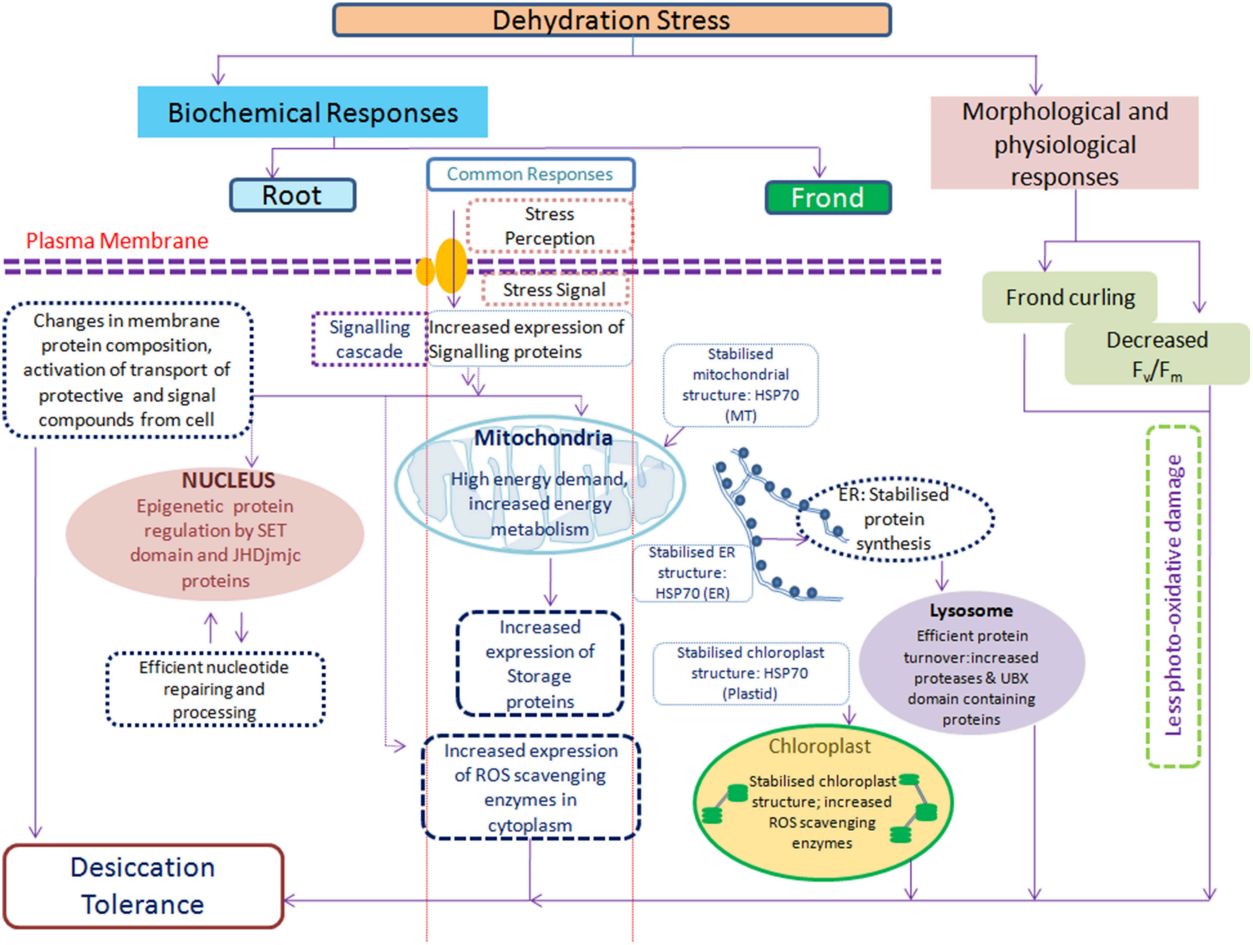
**Simplified model of ***S. bryopteris*** response to dehydration/rehydration based on physiological and proteomic data representing the collective actions of different mechanisms contributing toward the establishment of desiccation tolerance**.

This study showed that the proteome changes during dehydration and rehydration are very similar in roots and fronds as expected from a well-choreographed response from a resurrection plant (Figure 8). The challenge now is to discover how each of these groups actually moves the plant toward its goal of survival. A combination of transcriptomics, proteomics and metabolomics approaches would provide greater insight into how plants respond to dehydration stress. Knowledge gained from such systems biology approach will ultimately allow biotechnological approaches for the breeding of drought tolerant crops.

## Author contributions

FD and VP designed the experiment. FD and AP carried out the experimental studies. FD did data acquisition, analysis and interpretation of data. FD and VP wrote the paper. All authors have read and approved the manuscript.

### Conflict of interest statement

The authors declare that the research was conducted in the absence of any commercial or financial relationships that could be construed as a potential conflict of interest.

## Supplementary information

Supplementary Information 1 **Replicate gel images of root and fronds during dehydration and rehydrations (RI and RII)**.

Supplementary Information 2 **Percent volume values of protein spots in root and fronds of *S. bryopteris***.

Supplementary Information 3 **Detailed information of Mascot identified proteins**.

Supplementary Information 4 **Protein pilot identified proteins**.

Supplementary Information 5 **PCA analysis of root and frond proteins**.

Supplementary Information 6 **Figure of all protein spots differentially expressed during DE and RI and RII**.

## Abbreviations

PPFD: photosynthetic photon flux density
TCA: trichloroacetic acid
BME: Beta mercaptoethanol
DTT: Dithiothreitol
CHAPS: 3-[(3-cholamidopropyl)dimethylammonio]-1-propanesulfonate
ABA: Ammonium Bicarbonate
ACN: Acetonitrile
MALDI/TOF-TOF: Matrix assisted laser desorption ionization time of flight.
