# Supplementary material for: Organ Specific Proteomic Dissection of Selaginella bryopteris Undergoing Dehydration and Rehydration
Source: Front Plant Sci. 2016 Apr 8;7:425. doi: 10.3389/fpls.2016.00425 (PMC4824794; doi:10.3389/fpls.2016.00425)
Supplement: Supplementary Information 5 — PCA analysis of root and frond proteins. [file Image1.PDF]

# Gel Images of *S. bryopteris* Root

## Replicate gel Images of S. bryopteris ROOT: Control

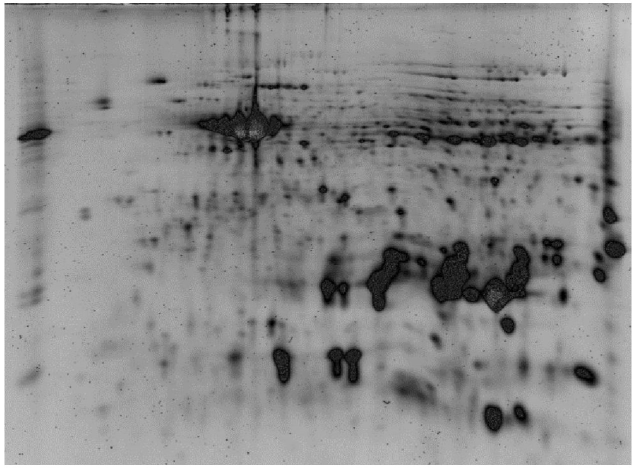

Control A

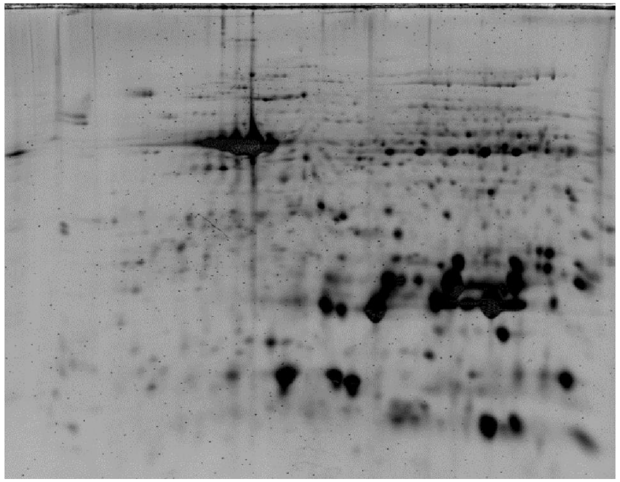

Control B

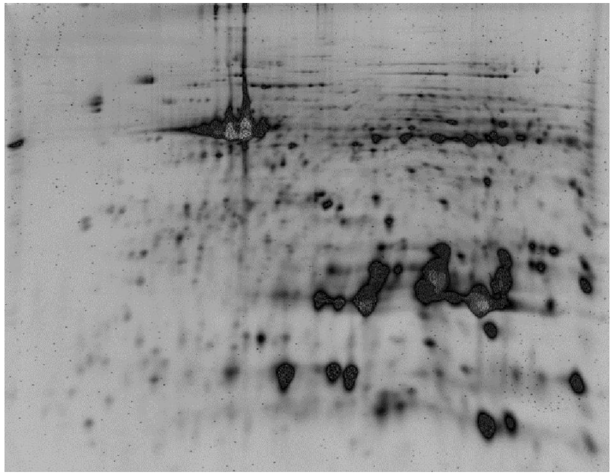

Control C

**Replicate gel Images of S. bryopteris ROOT; Dehydrated**

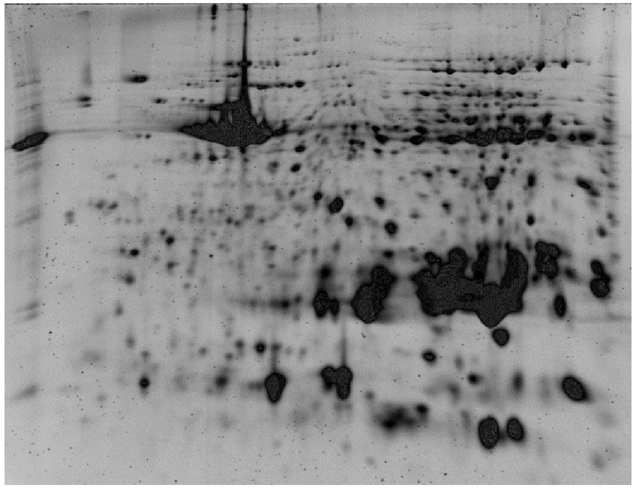

Dehydrated A

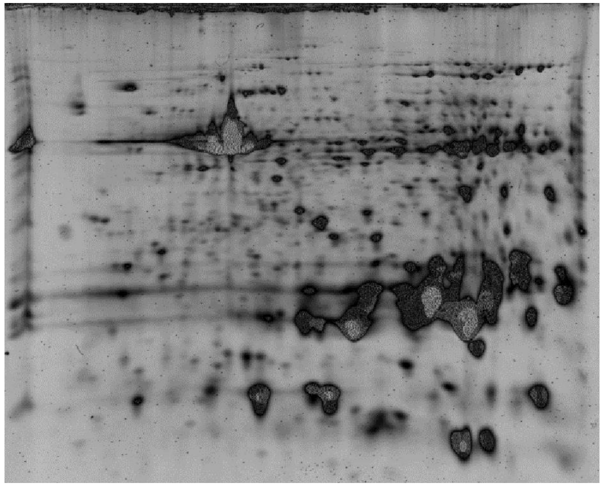

Dehydrated B

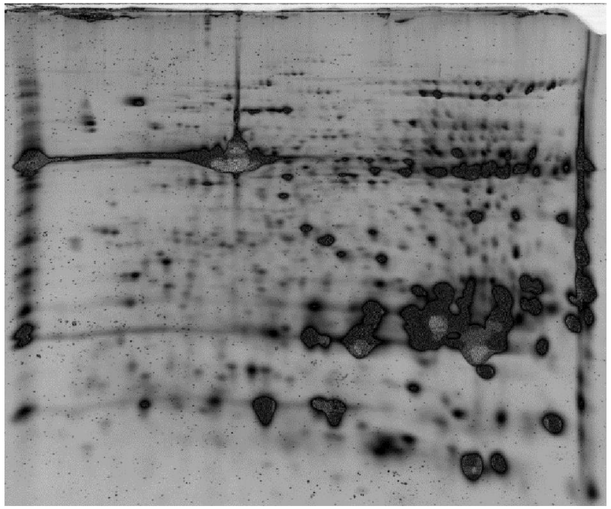

Dehydrated C

**Replicate gel Images of S. bryopteris ROOT: First Rehydration (RI)**

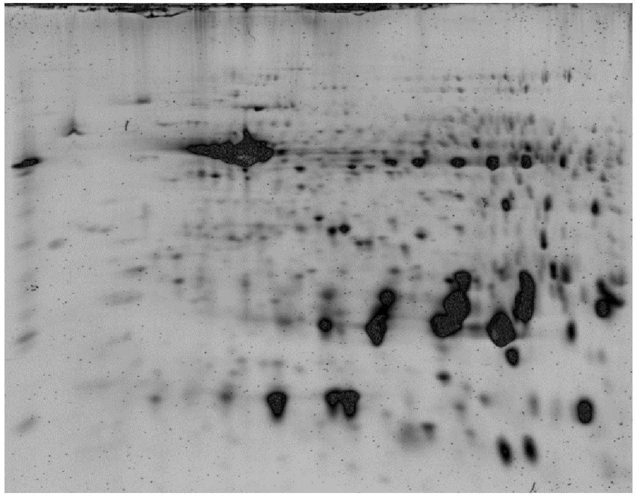

RI-A

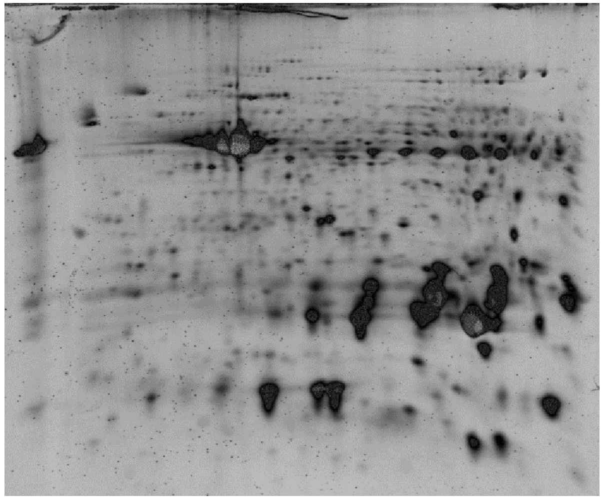

RI-B

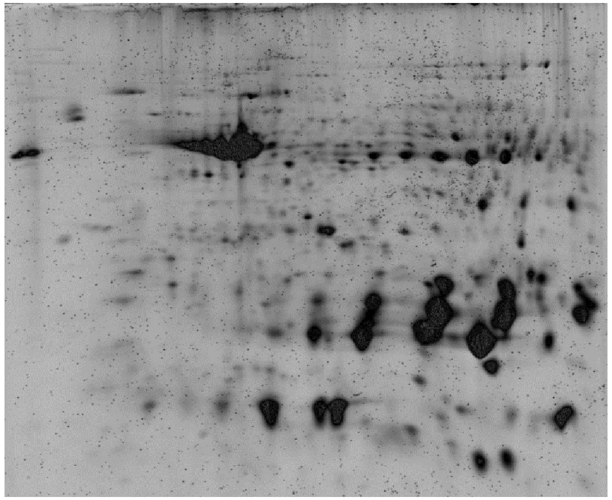

RI-C

## Replicate gel Images of S. bryopteris ROOT: Second Rehydration RII

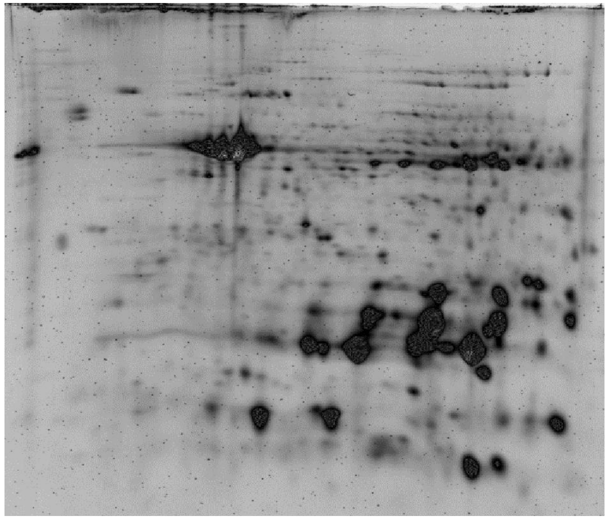

RII-A

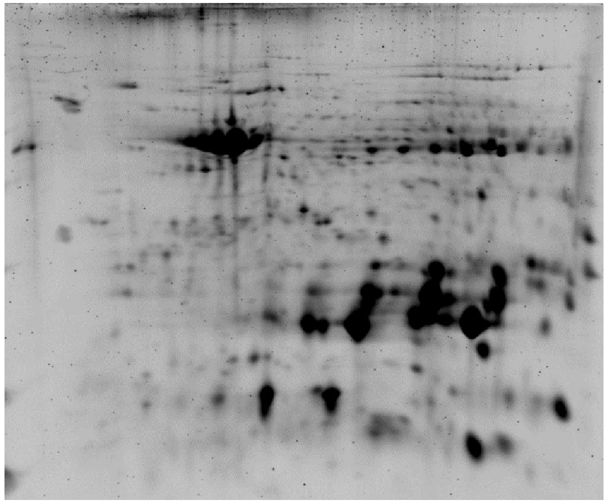

RII-B

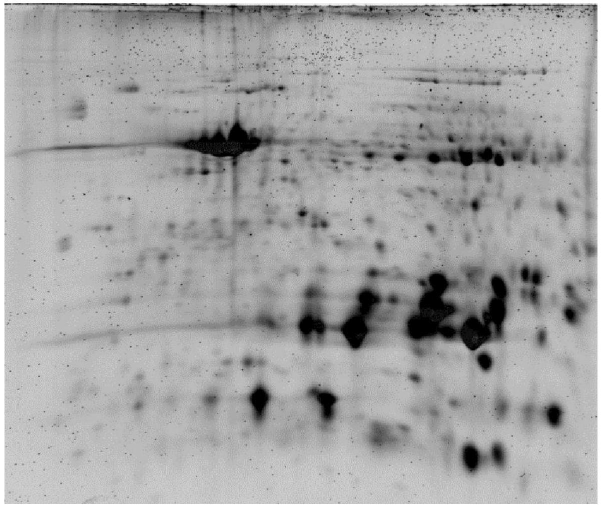

RII-C

# Gel Images of S. bryopteris Fronds

## Replicate gel Images of S. bryopteris Frond: Control

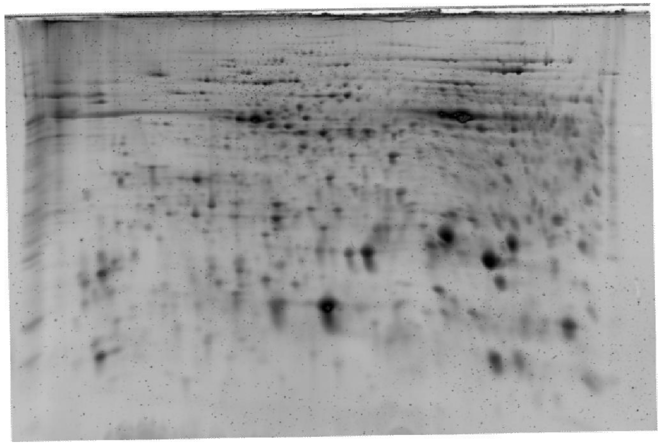

Control A

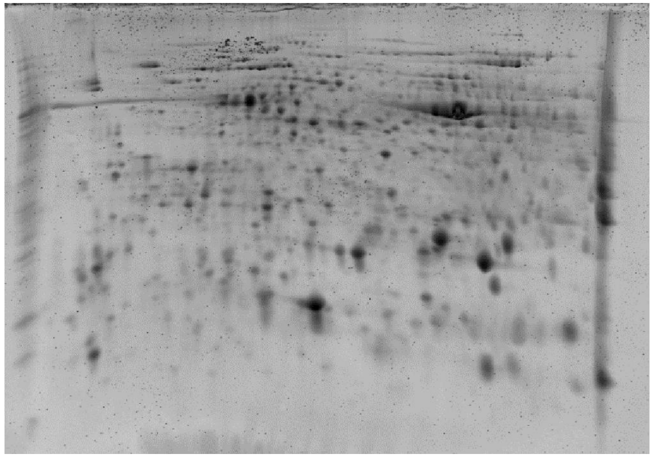

Control B

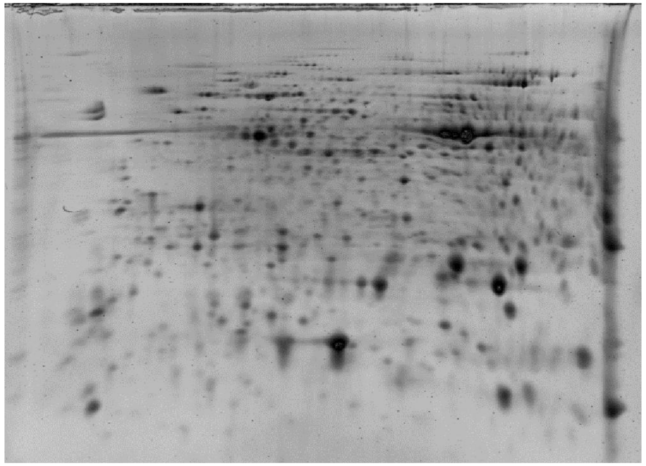

Control C

**Replicate gel Images of S. bryopteris Frond: Dehydrated**

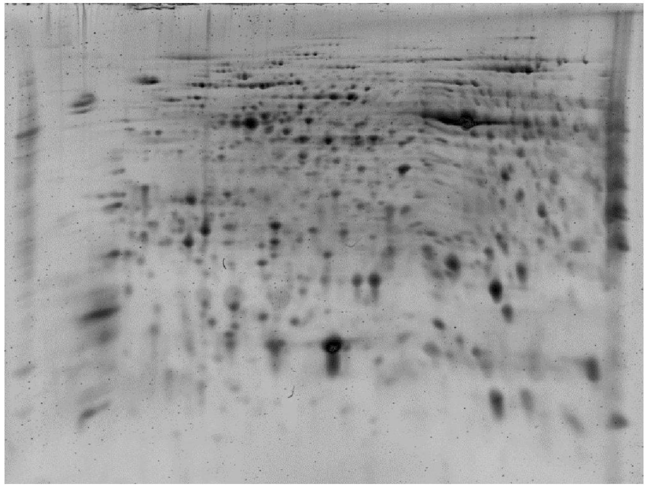

Dehydrated A

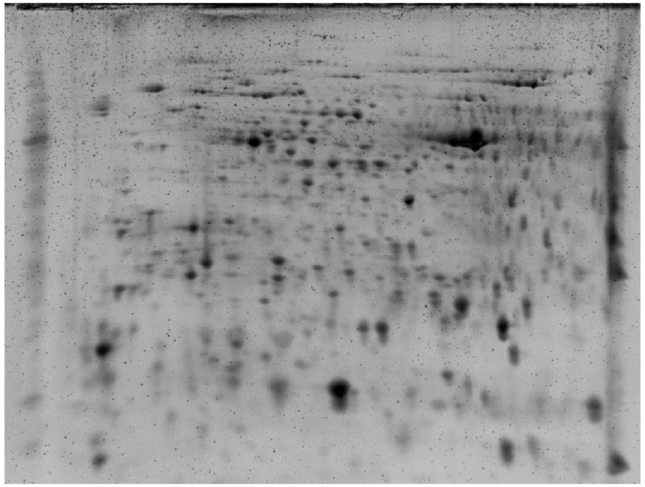

Dehydrated B

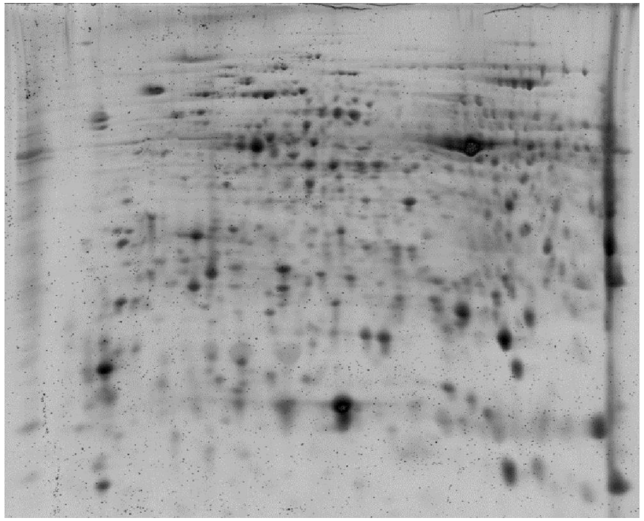

Dehydrated C

**Replicate gel Images of S. bryopteris Frond: First Rehydration RI**

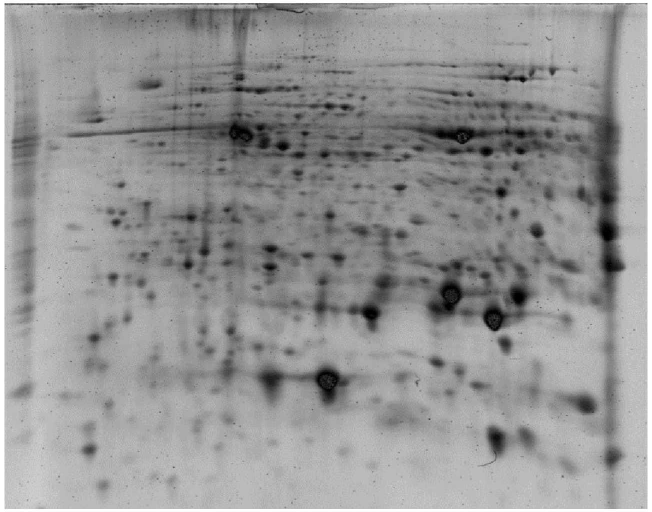

RI-A

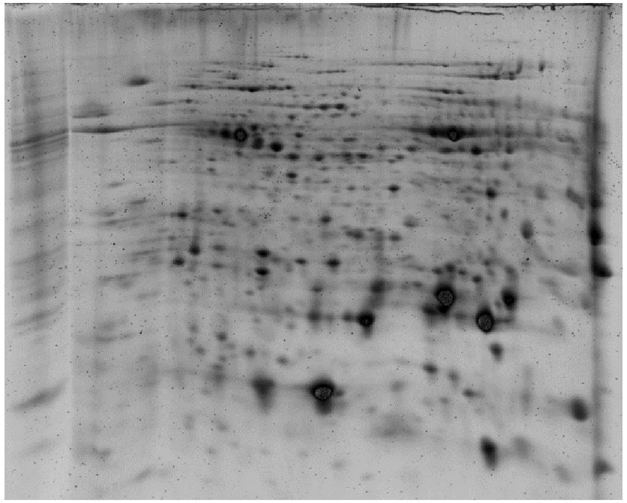

RI-B

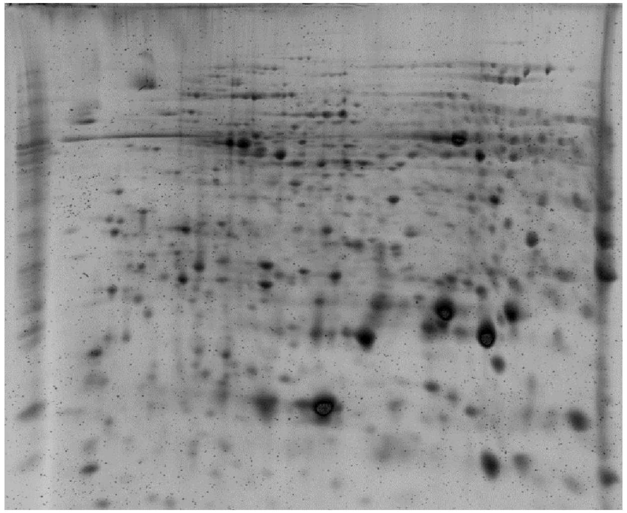

RI-C

**Replicate gel Images of S. bryopteris Frond: Second Rehydration RII**

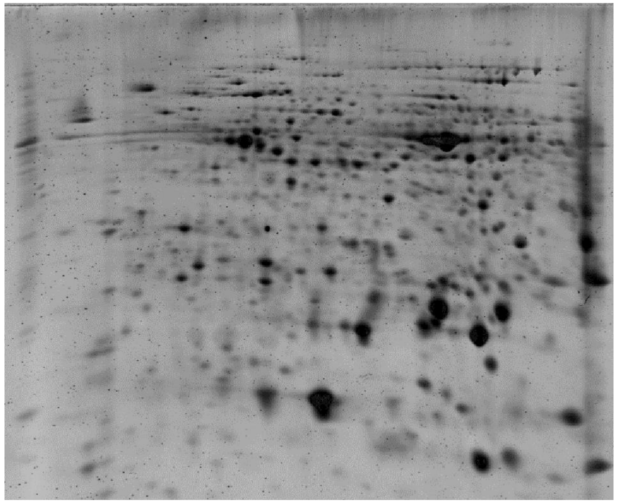

RII-A

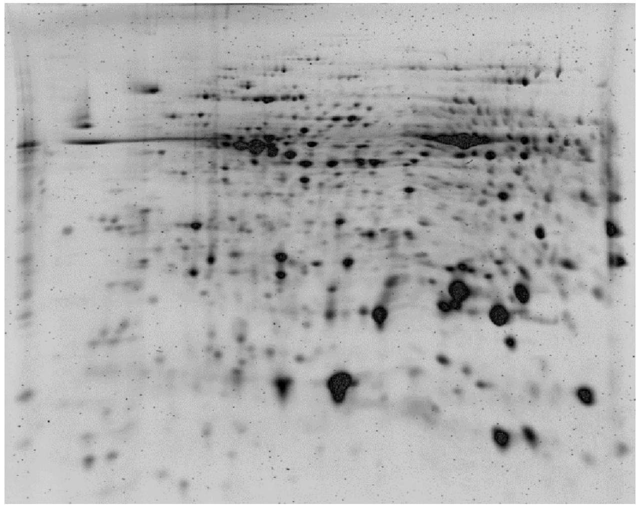

RII-B

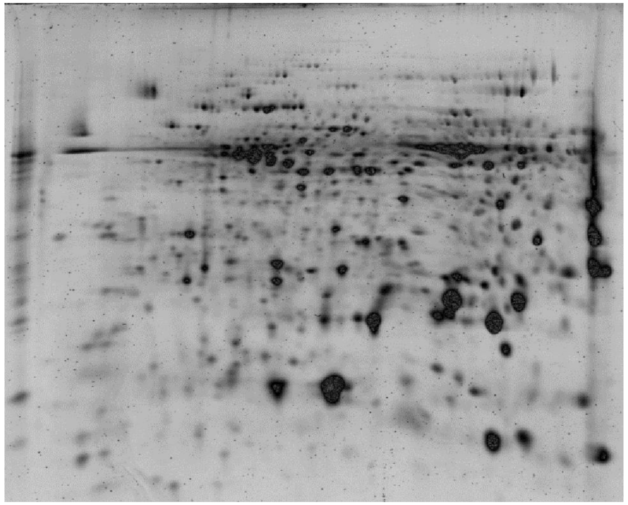

RII-C
