# Supplementary material for: Organ Specific Proteomic Dissection of Selaginella bryopteris Undergoing Dehydration and Rehydration
Source: Front Plant Sci. 2016 Apr 8;7:425. doi: 10.3389/fpls.2016.00425 (PMC4824794; doi:10.3389/fpls.2016.00425)
Supplement: Supplementary Information 6 — Figure of all protein spots differentially expressed during DE and RI and RII. [file Table1.DOC]

**Table S1** Diffrentially expressed proteins and their % volume values of *S. bryopteris* roots during desiccation and rehydration (RI and RII).

| **SpotNo** | **Protein expression** | **Histogram** | **Identified Protein** |
| --- | --- | --- | --- |

Con Des R-I R-II


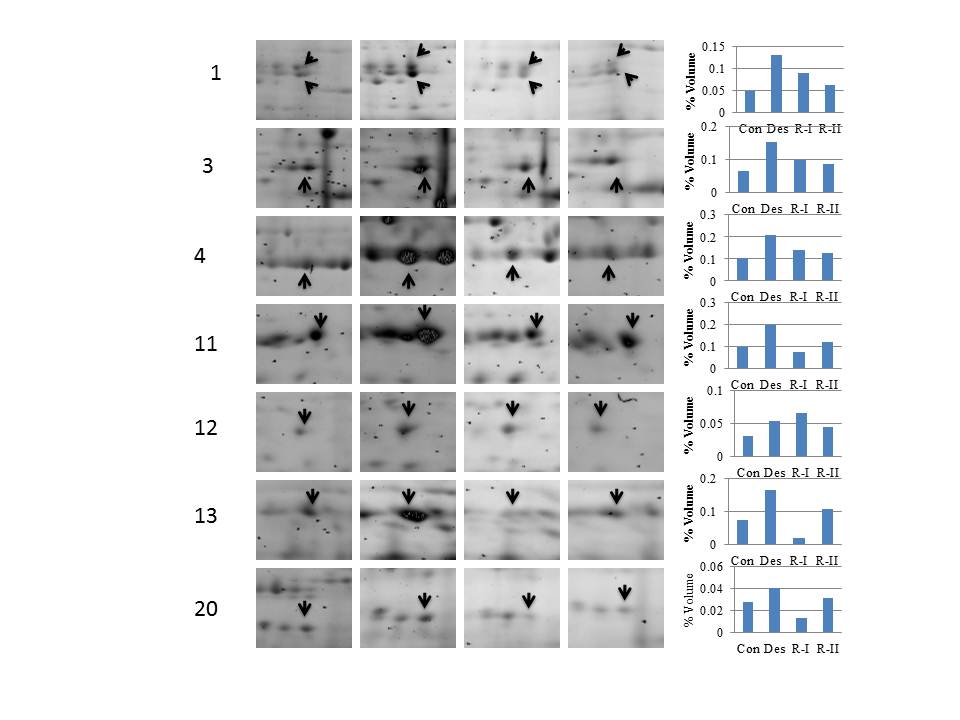


nutrient reservoir, putative

Peptide chain release factor 1

nucleoside-triphosphatase/ nucleotide binding protein

Hsc70

heat shock protein, putative

similar to S. cerevisiae PTR2 gene,

LAS1-like family protein


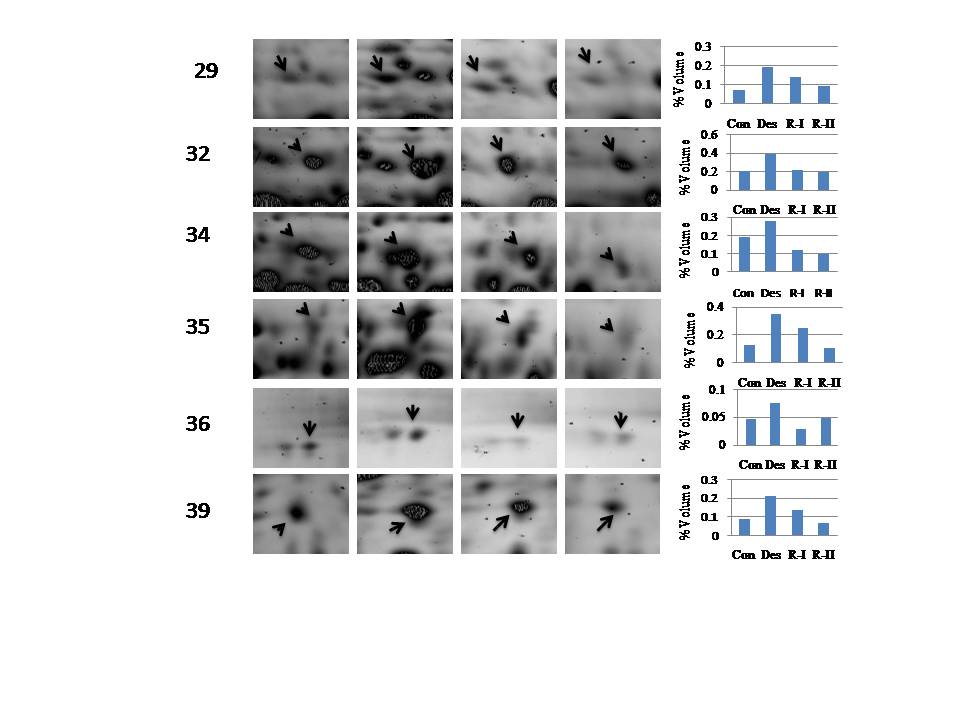


Enolase

Enolase

ALDH11A3

tau class glutathione S-transferase

putative DNA repair protein RAD23-1

RecName: Full=Actin

Continued…

| **Spot No** | **Protein expression** | **Histogram** | **Identified Protein** |
| --- | --- | --- | --- |

Con Des R-I R-II


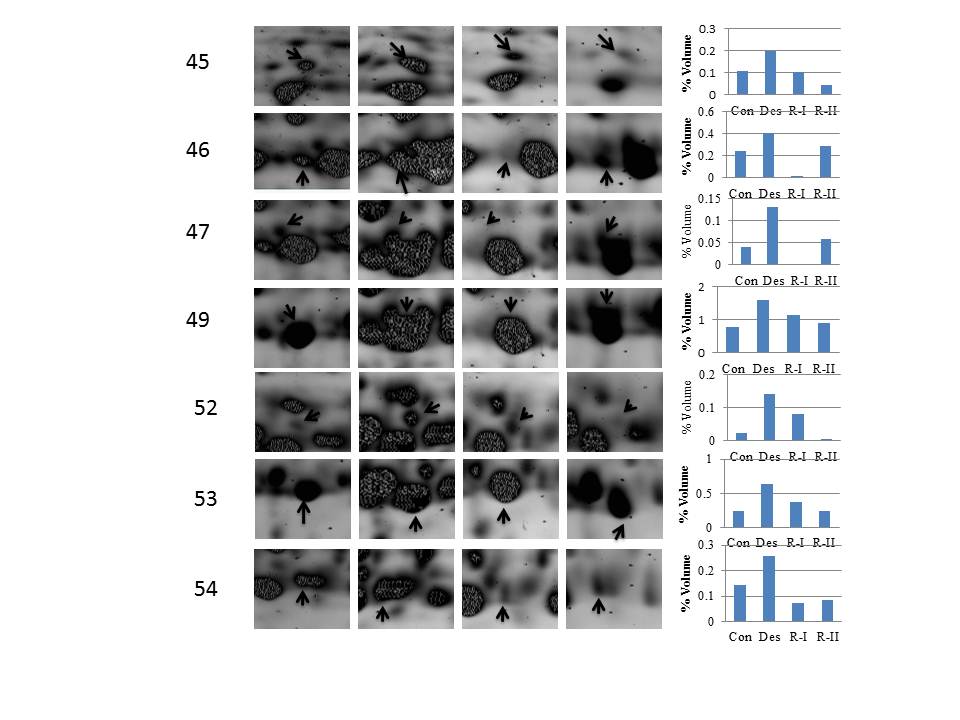

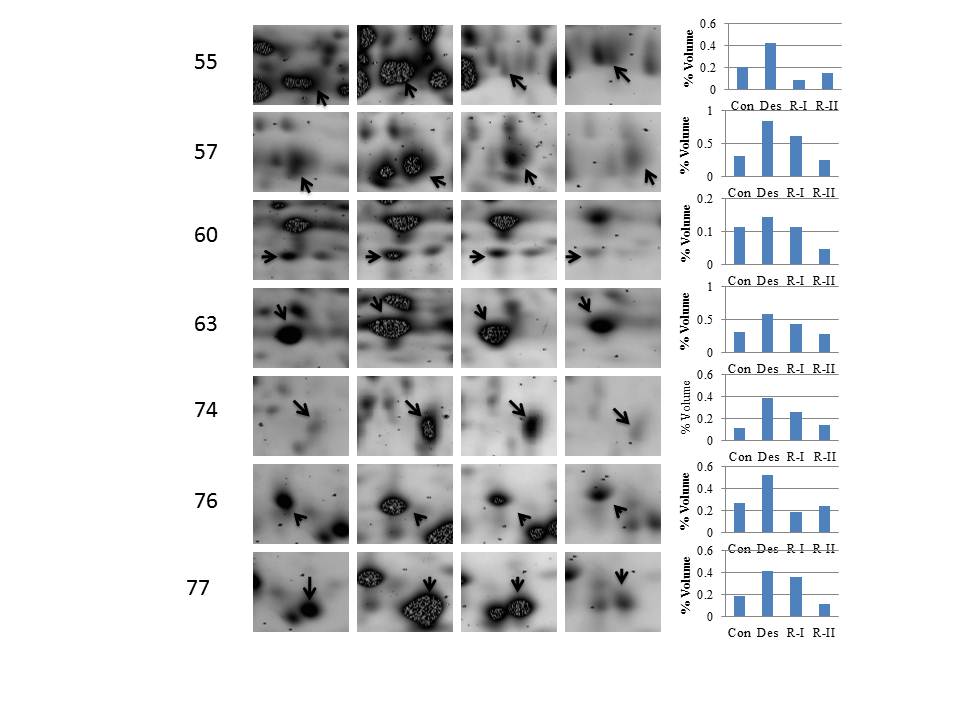


phospholipase A1-IIgamma-like [Solanum lycopersicum]

RNA binding protein, putative

predicted protein

maturase K

ATPase subunit

JmjC domain-containing histone demethylation protein 1

cupin family protein

aerobactin synthetase

short-chain dehydrogenase, putative

glucan endo-1,3-alpha-glucosidase Agn1

RmlC-like cupin

Glucose and ribitol dehydrogenase

envelope protein gp90

ATP-binding cassette transporter, subfamily C

Continued…

| **Spot No** | **Protein expression** | **Histogram** | **Identified Protein** |
| --- | --- | --- | --- |

Con Des R-I R-II


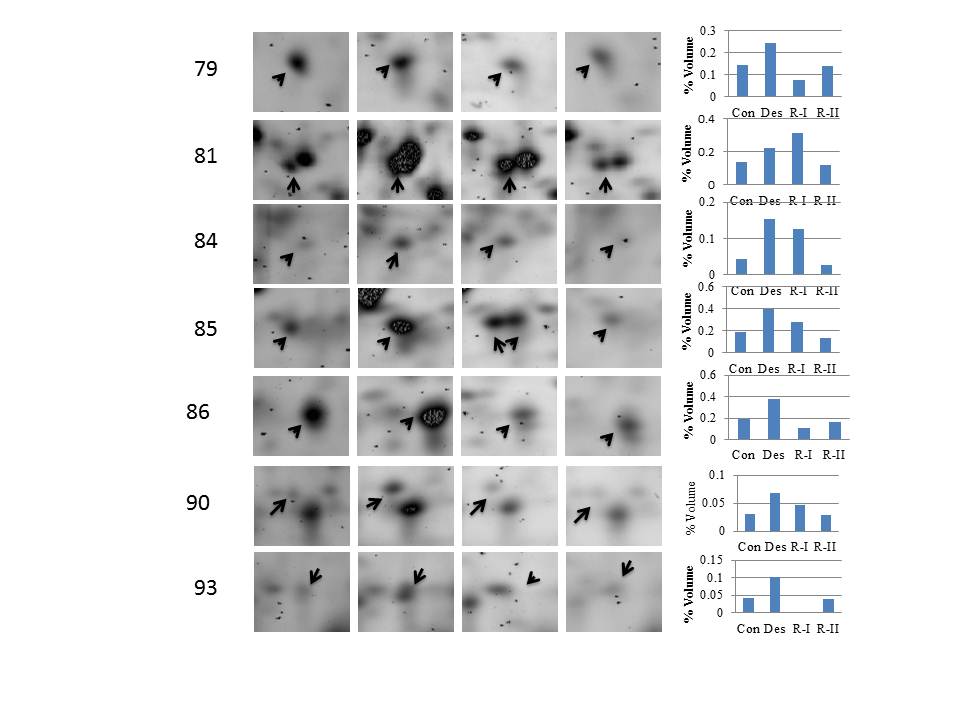

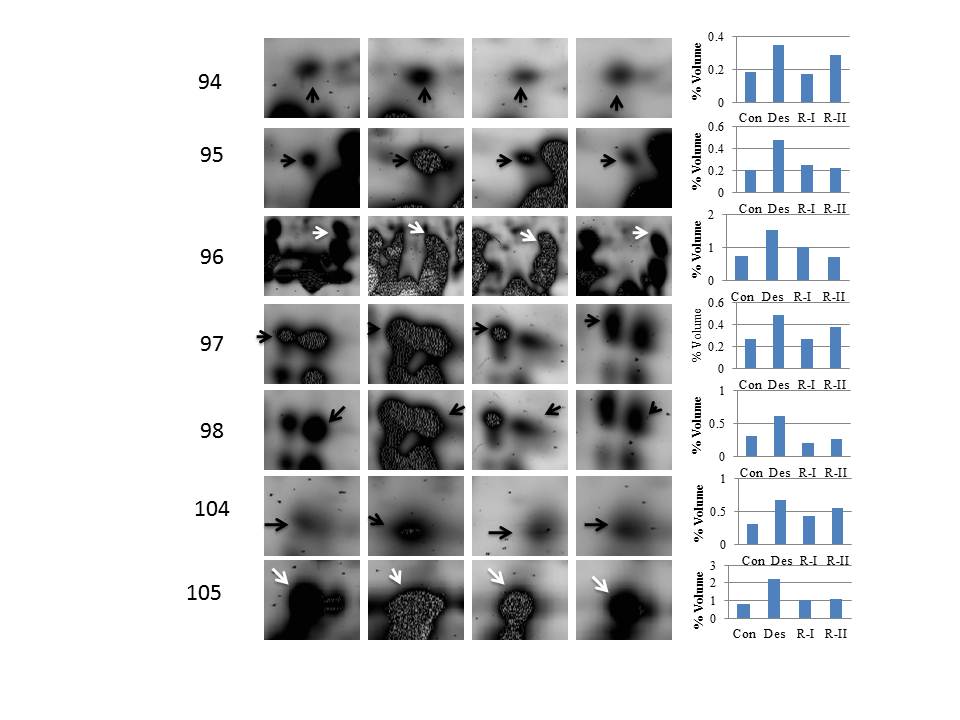


strumpellin

lactoylglutathione lyase, putative

putative protein phosphatase 2C 10

ketose-bisphosphate aldolase class-II-like protein

pentatricopeptide repeat-containing protein

14-3-3d protein

ATPase alpha subunit

pyrimidine-specific ribonucleoside hydrolase rihA

cupin family protein

aminoacyl-t-RNA synthetase

related to JHD1-JmjC domain family histone demethylase specific for H3-K36

pentatricopeptide repeat-containing protein

related to JHD1-JmjC domain family histone demethylase specific for H3-K36

Glutelin type-A

Continued…

| **Spot No** | **Protein expression** | **Histogram** | **Identified Protein** |
| --- | --- | --- | --- |

Con Des R-I R-II


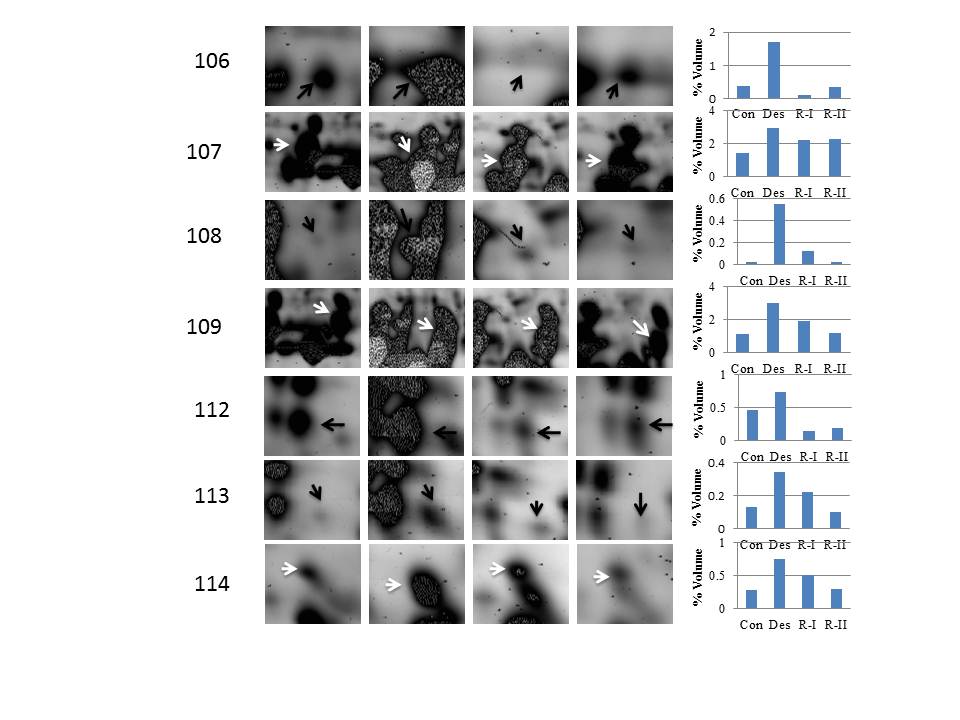

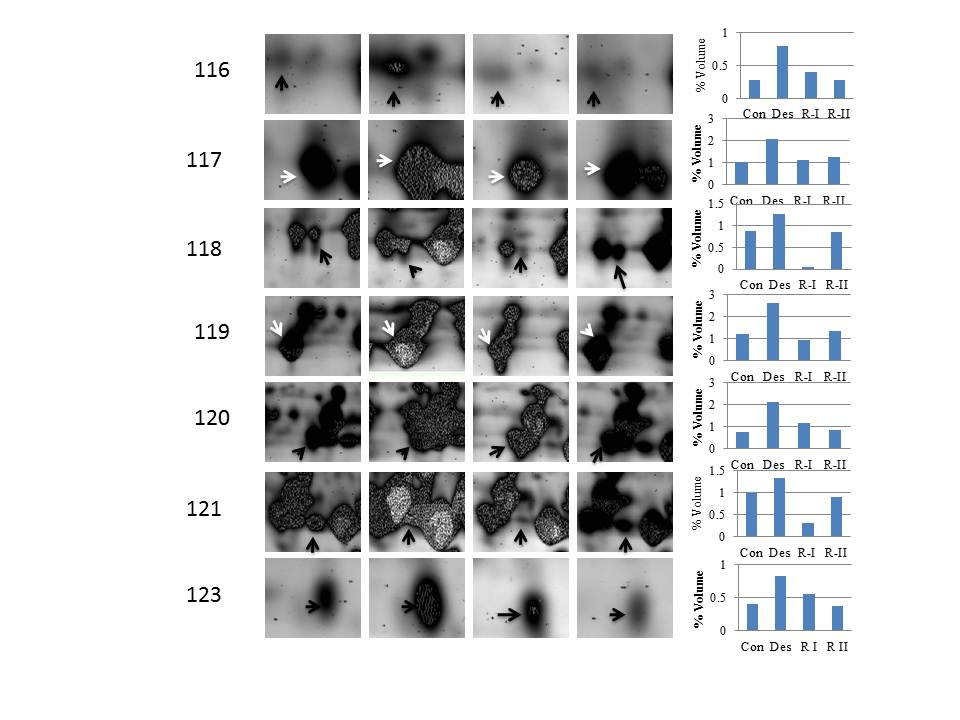


probable LRR receptor-like serine/threonine-protein kinase At1g29720-like [Vitis vinifera]

glutathione S-transferase-like protein

ATP binding protein, putative

Quinonprotein alcohol dehydrogenase-like

RmlC-like cupin

Thioredoxin-like protein

serine carboxypeptidase

family protein

maturase K

hypothetical protein SELMODRAFT_428082

Glutelin type-A

Putative hemolysin

hypothetical protein SELMODRAFT_159799

cupin family protein

SET domain protein 35

Continued…

| **Spot No** | **Protein expression** | **Histogram** | **Identified Protein** |
| --- | --- | --- | --- |

Con Des R-I R-II


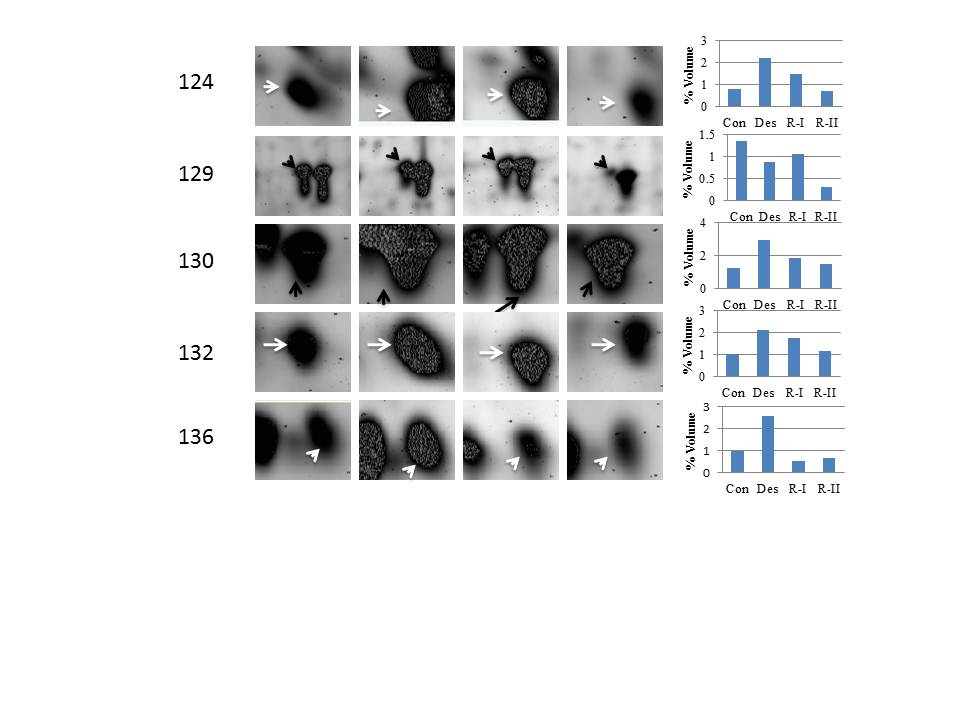


DMI1 protein

ankyrin repeat-

containing protein

Nucleotidyltransferase

family protein, putative

UBX domain-

containing protein

leucine-rich repeat

family protein

**Table S2.** Diffrentially expressed proteins with their %volume values in *Selaginella* fronds during desiccation and rehydration.

| **Spot No** | **Protein expression** | **Histogram** | **Identified Protein** |
| --- | --- | --- | --- |

[Con Des R-I R-II](http://blast.ncbi.nlm.nih.gov/Blast.cgi#alnHdr_545366438)


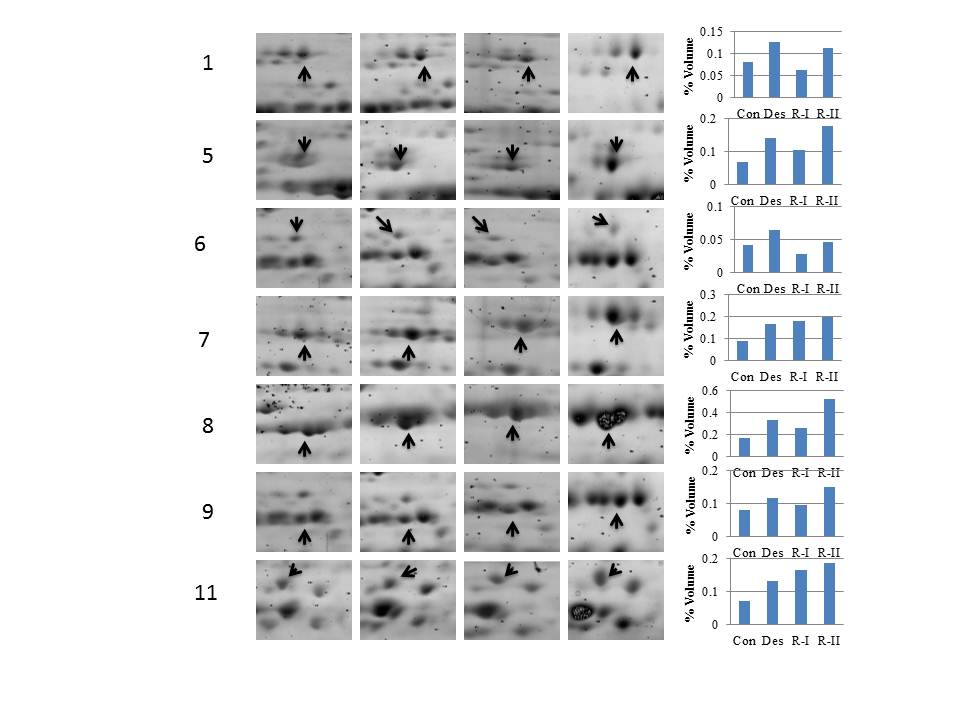

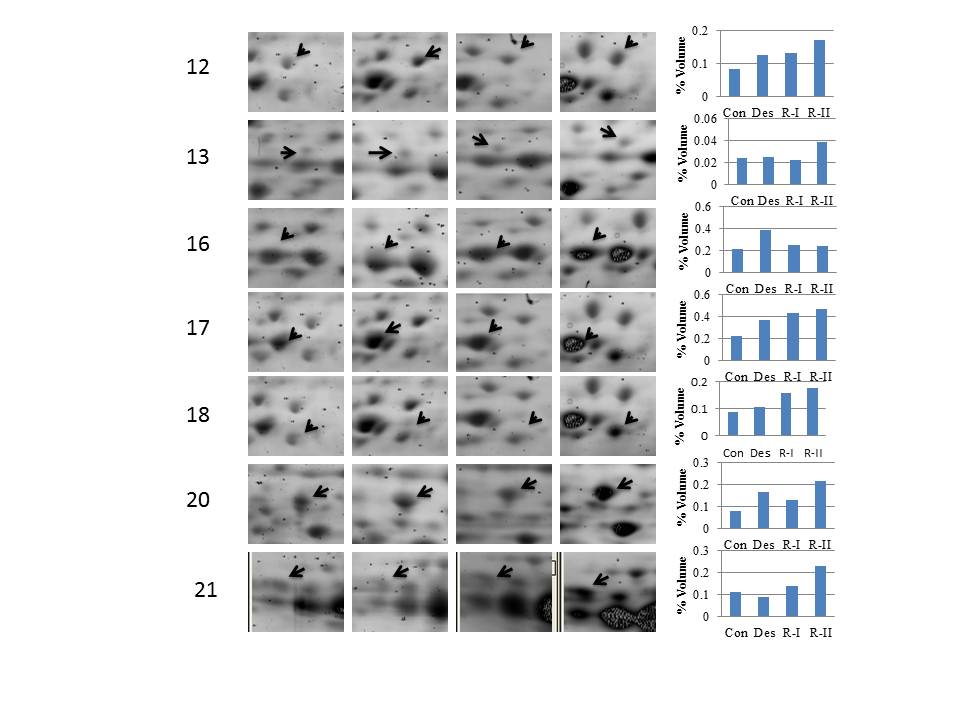


[Putative spindle disassembly related protein CDC48](http://blast.ncbi.nlm.nih.gov/Blast.cgi#alnHdr_545366438)

[81kDa heat-shock protein](http://blast.ncbi.nlm.nih.gov/Blast.cgi#alnHdr_545366438)

[heat shock protein, putative](http://blast.ncbi.nlm.nih.gov/Blast.cgi#alnHdr_545366438)

[stromal 70 kDa heat shock-related protein](http://blast.ncbi.nlm.nih.gov/Blast.cgi#alnHdr_545366438)

[luminal binding protein](http://blast.ncbi.nlm.nih.gov/Blast.cgi#alnHdr_545366438)

[PREDICTED: heat shock 70 kDa protein, mitochondrial-like](http://blast.ncbi.nlm.nih.gov/Blast.cgi#alnHdr_545366438)

[PREDICTED: heat shock 70 kDa protein, mitochondrial-like](http://blast.ncbi.nlm.nih.gov/Blast.cgi#alnHdr_545366438)

[membrane AAA-metalloprotease](http://blast.ncbi.nlm.nih.gov/Blast.cgi#alnHdr_545366438)

[DAO-domain-containing protein](http://blast.ncbi.nlm.nih.gov/Blast.cgi#alnHdr_545366438)

[ATP synthase CF1 alpha chain](http://blast.ncbi.nlm.nih.gov/Blast.cgi#alnHdr_545366438)

[DAO-domain-containing protein…..](http://blast.ncbi.nlm.nih.gov/Blast.cgi#alnHdr_545366438)

[Chaperonin CPN60-like protein](http://blast.ncbi.nlm.nih.gov/Blast.cgi#alnHdr_545366438)

[ATP synthase CF1 alpha chain](http://blast.ncbi.nlm.nih.gov/Blast.cgi#alnHdr_545366438)

[beta-tubulin](http://blast.ncbi.nlm.nih.gov/Blast.cgi#alnHdr_545366438)

Continued

| **Spot No** | **Protein expression** | **Histogram** | **Identified Protein** |
| --- | --- | --- | --- |

ConDes R-I R-II


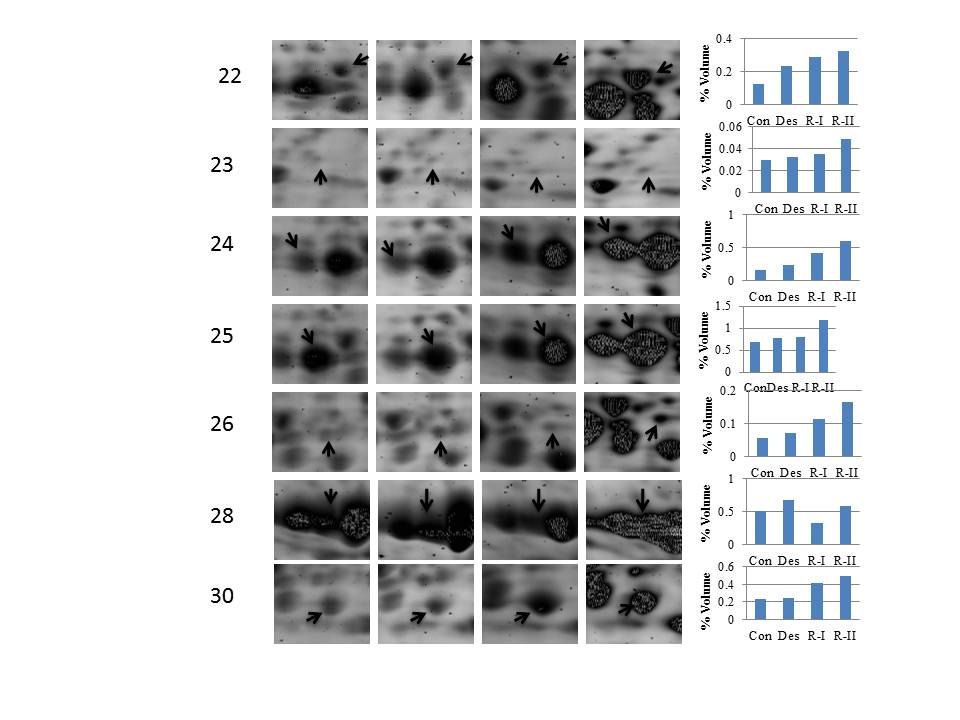

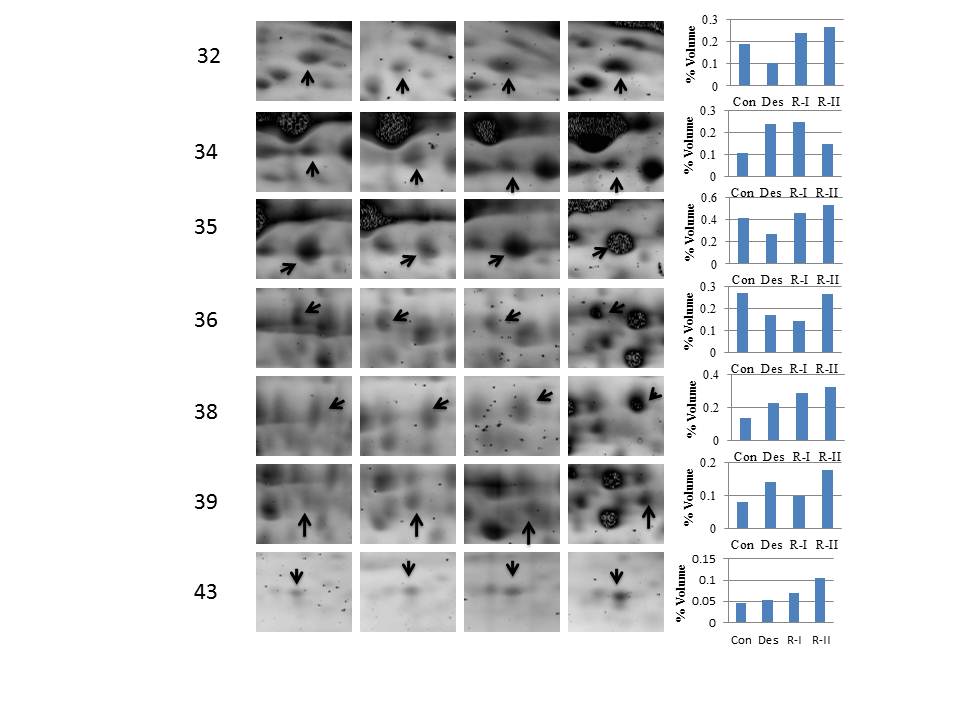


 mitochondrial F1-

ATPase beta subunit

ribulose-1,5-bisphosphate carboxylase/oxygenase

hypothetical protein SELMODRAFT_418602

ATP synthase subunit beta, mitochondrial

ATP synthase subunit beta, mitochondrial

ribulose-1,5-bisphosphate carboxylase/oxygenase

RecName: Full=Legumin A2

cupin family protein

chloroplast elongation factor tub

cupin family protein

ribulose-1,5-bisphosphate carboxylase/oxygenase

ribulose bisphosphate carboxylase, partial (chloroplast)

ribosome biogenesis serine/threonine protein kinase (Prp4), putative [*Aspergillus fumigatus* A1163]

Late embryogenesis abundant protein Lea14-A, putative

Continued…

| **Spot No** | **Protein expression** | **Histogram** | **Identified Protein** |
| --- | --- | --- | --- |

Con Des R-I R-II


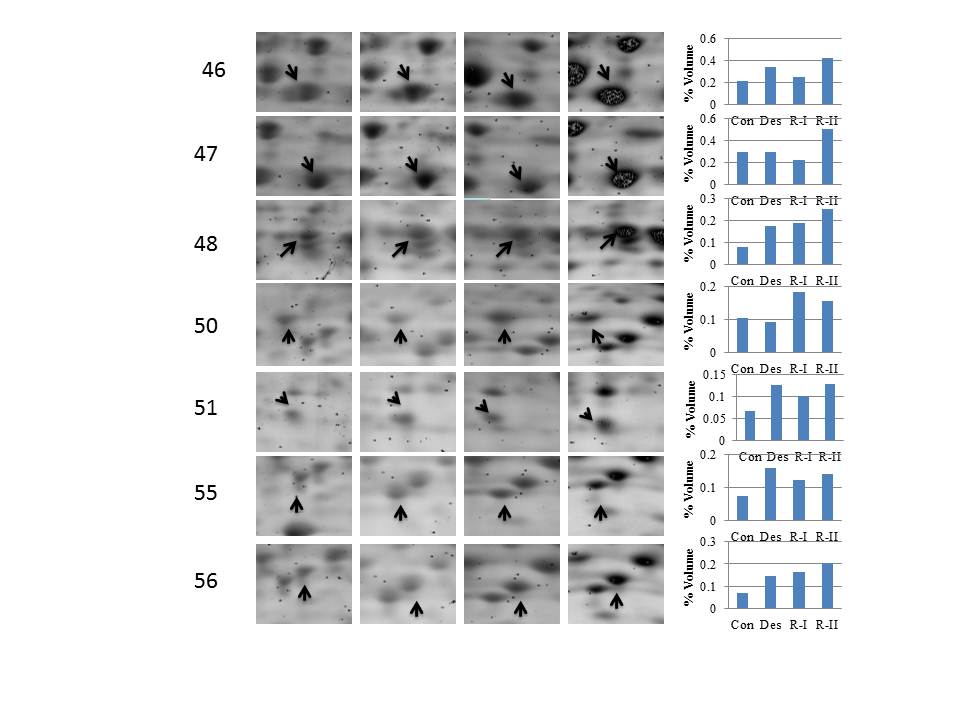

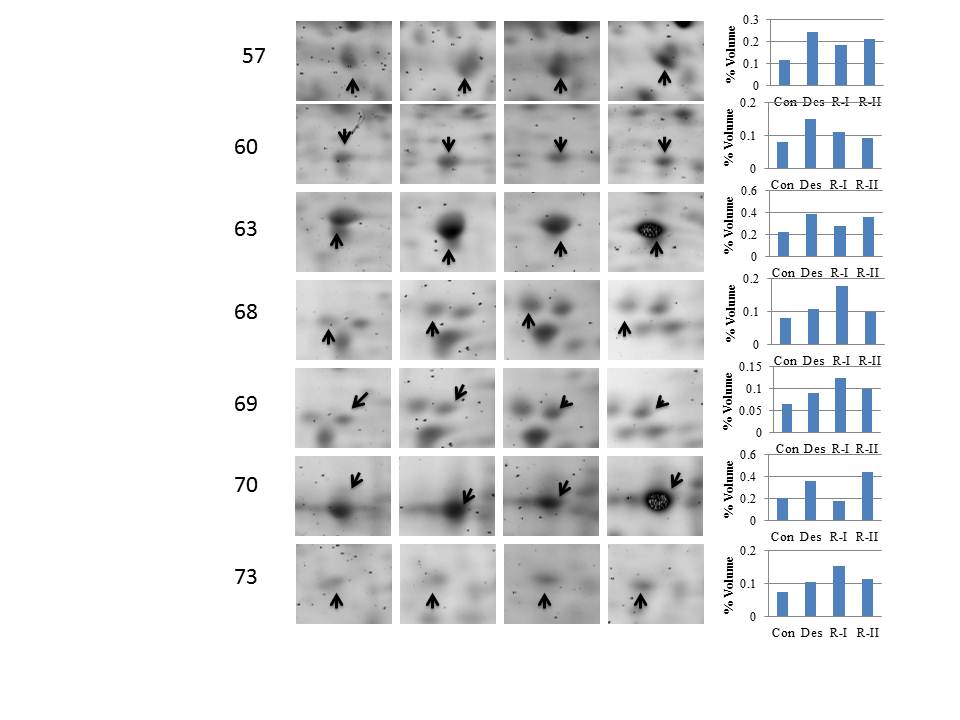


actin

rubisco activase

monodehydroascorbate reductase

RmlC-like cupin

sedoheptulose-1,7-bisphosphatase, chloroplast, putative

phosphoglycerate kinase, chloroplast

phosphoglycerate kinase

GDP-mannose 3,5-epimerase

Fructose-bisphosphate aldolase

Fructose-bisphosphate

aldolase

cysteine protease

cysteine protease

Oxygen-evolving enhancer protein 1, chloroplastic

centrosomal protein of 44 kDa

Continued…

| **Spot No** | **Protein expression** | **Histogram** | **Identified Protein** |
| --- | --- | --- | --- |

Con Des R-I R-II


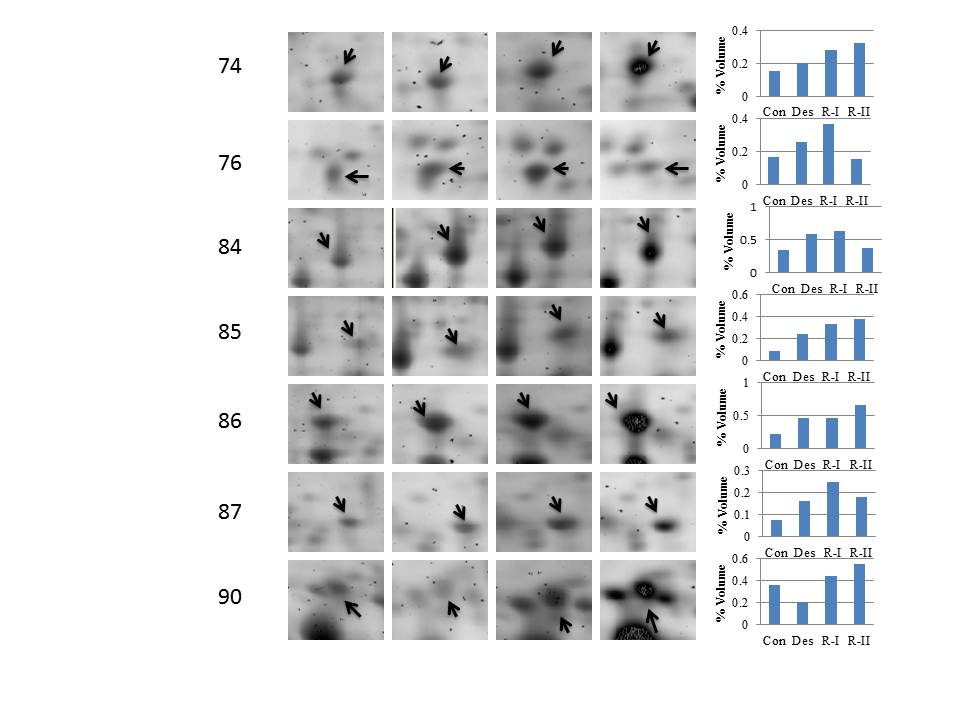

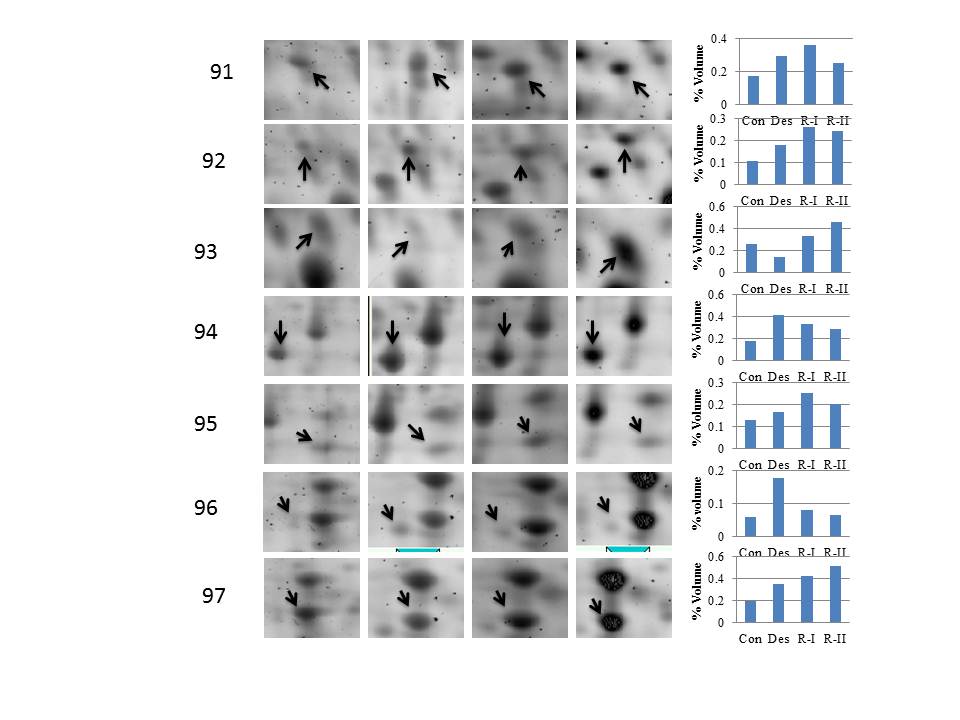


lactoylglutathione lyase,

chloroplast 29 kDa ribonucleoprotein

chlorophyll a/b binding protein of LHCII type I

Ubiquitin thioesterase OTU1

Pentatricopeptide repeat-containing protein

ferritin, chloroplast precursor

related to JHD1-JmjC domain family histone demethylase specific for H3-K36

trithorax-like protein, histone-lysine N-methyltransferase

ascorbate peroxidase

glutathione S-transferase-like protein

light-harvesting chlorophyll a/b-binding protein of photosystem II

Proteasome subunit beta type-3

pentatricopeptide repeat-containing protein

tau class glutathione S-transferase

Continued..

| **Spot No** | **Protein expression** | **Histogram** | **Identified Protein** |
| --- | --- | --- | --- |

Con Des R-I R-II


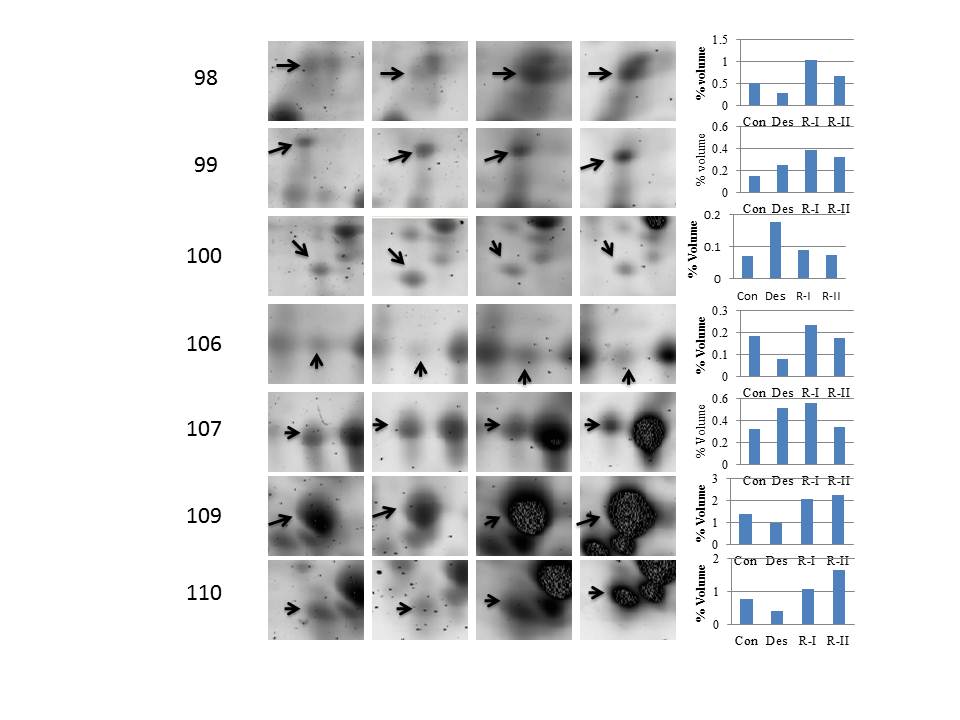

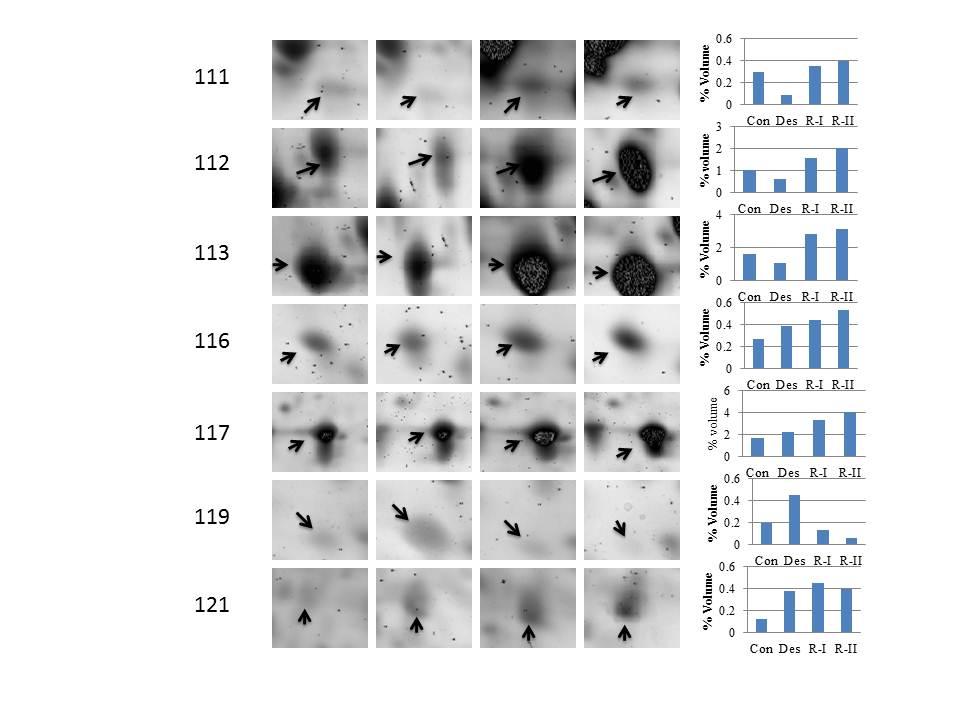


dehydration responsive element binding protein

ATP binding protein, putative

2-Cys-peroxiredoxin

predicted protein

oxygen-evolving

enhancer protein 2

hypothetical protein CHLREDRAFT_150252

maturase K

maturase K

Quinonprotein alcohol dehydrogenase-like

maturase K

copper-zinc superoxide dismutase

ankyrin repeat-containing protein

nucleoside diphosphate kinase

Glucose and ribitol dehydrogenase

Continued…

| **Spot No** | **Protein expression** | **Histogram** | **Identified Protein** |
| --- | --- | --- | --- |

Con Des R-I R-II


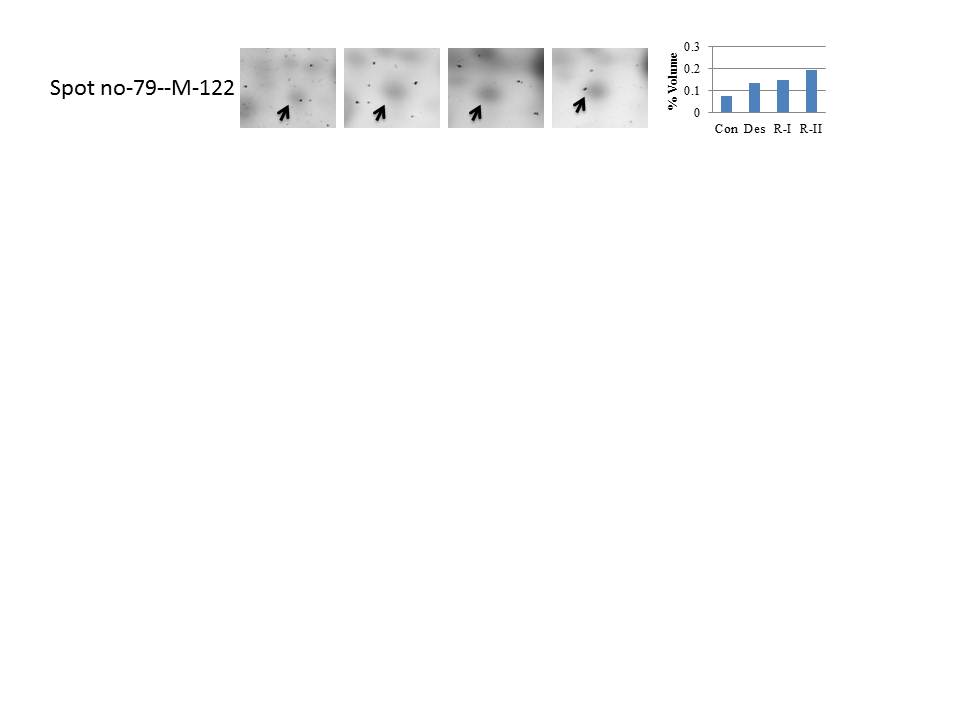


Beta-conglycinin,

alpha chain; Flags: Precursor

**Table S2 B**. 3D images of some protein spots of *S. bryopteris* fronds retrieved from Image Mater Platinum 2D- differentially expressed during desiccation and recovery

| **Spot No** | **Protein expression** | **Histogram** | **Identified Protein** |
| --- | --- | --- | --- |

Con Des R-I R-II


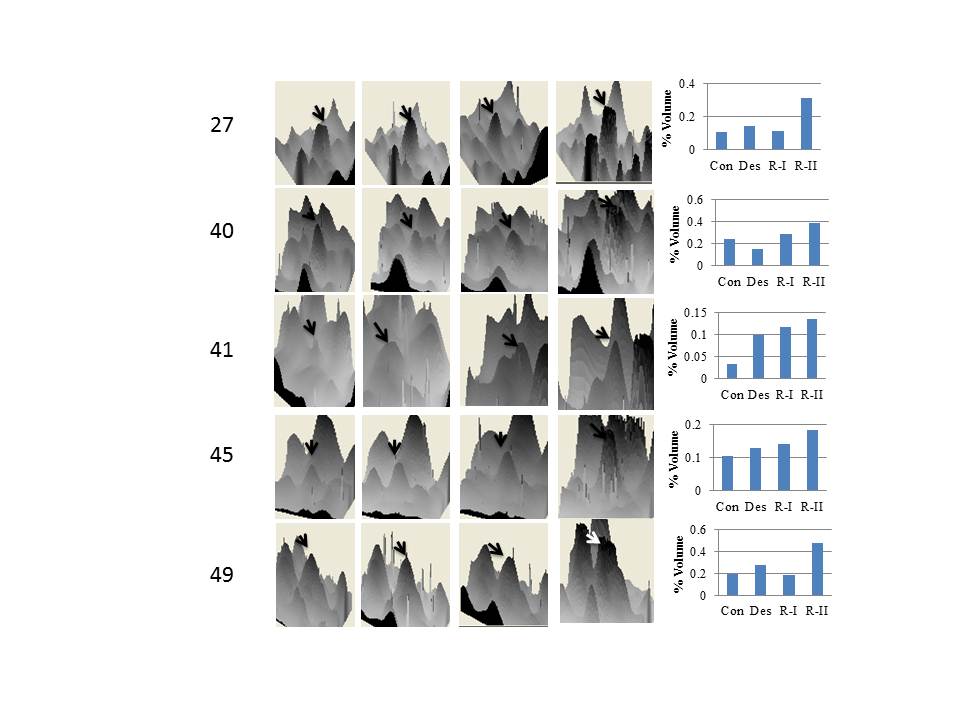

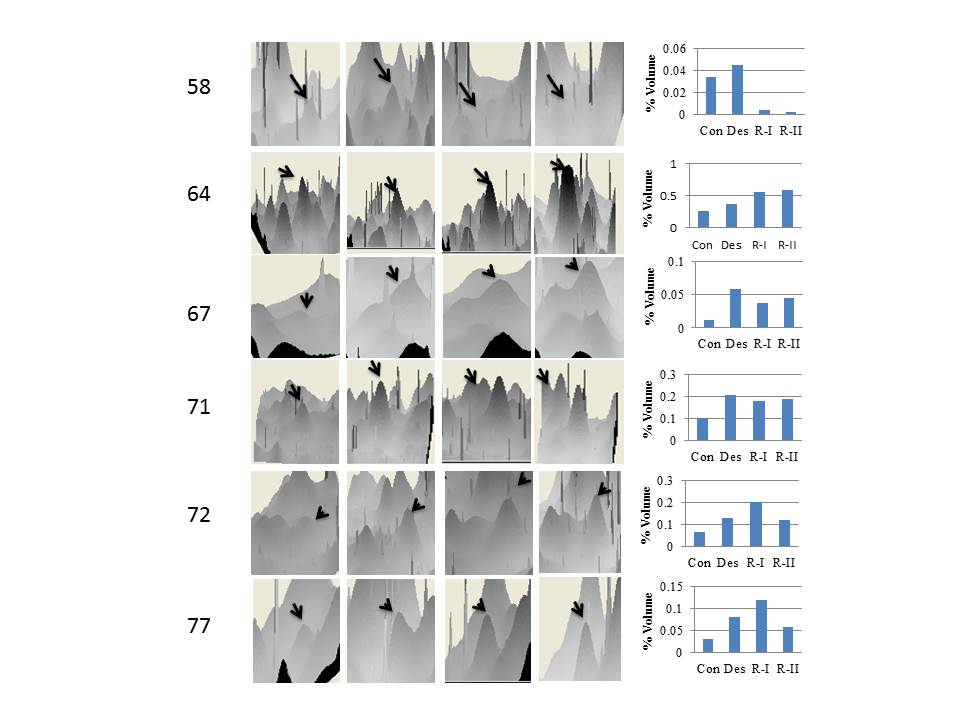


ATP synthase subunit beta, mitochondrial

Transcription

initiation factor TFIID, subunit TAF1

cupin family protein

putative peroxidase

plastidic glutamine

synthetase precursor

predicted protein

dormancy related protein, putative

Omega-amidase NIT2

dessication-related protein, putative

PREDICTED: desiccation-related protein PCC13-62-like

14-3-3d protein

Continued…

| **Spot No** | **Protein expression** | **Histogram** | **Identified Protein** |
| --- | --- | --- | --- |

Con Des R-I R-II


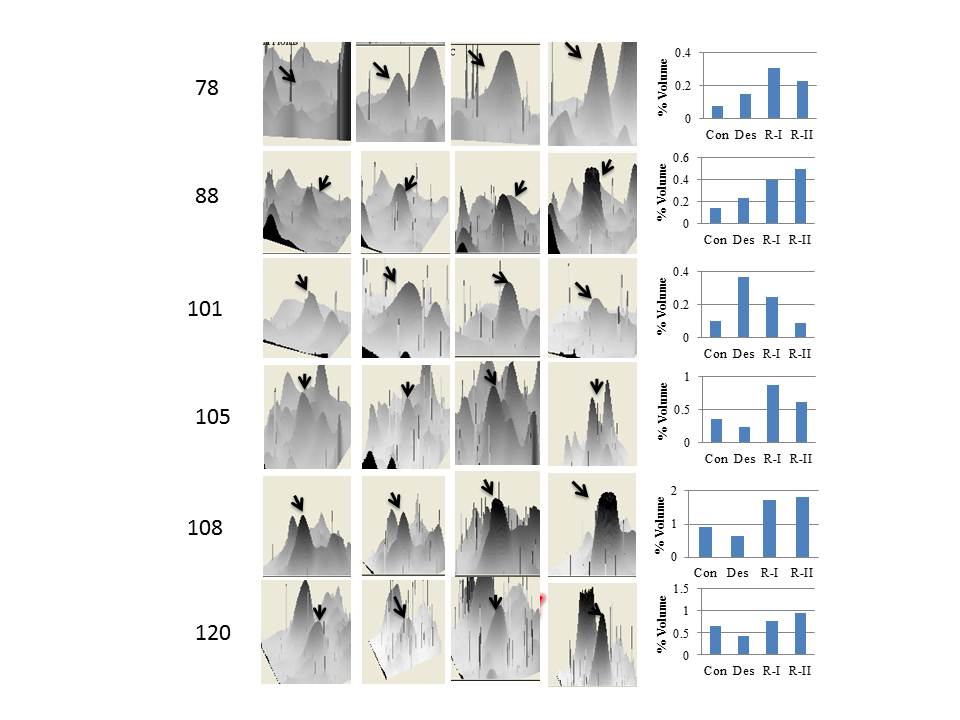


Beta-conglycinin, alpha chain; Flags: Precursor

Paf1 complex protein

cysteine protease

maturase K

maturase K

hypothetical protein SELMODRAFT_444247

|  |  |
| --- | --- |
|  |  |
|  |  |
